# LUBAC controls chromosome alignment by targeting CENP-E to attached kinetochores

**DOI:** 10.1038/s41467-018-08043-7

**Published:** 2019-01-17

**Authors:** Min Wu, Yan Chang, Huaibin Hu, Rui Mu, Yucheng Zhang, Xuanhe Qin, Xiaotao Duan, Weihua Li, Haiqing Tu, Weina Zhang, Guang Wang, Qiuying Han, Ailing Li, Tao Zhou, Kazuhiro Iwai, Xuemin Zhang, Huiyan Li

**Affiliations:** 1 State Key Laboratory of Proteomics, National Center of Biomedical Analysis, 100850 Beijing, China; 2 Department of Radiation Oncology, National Cancer Center/Cancer Hospital, Chinese Academy of Medical Sciences and Peking Union Medical College, 100021 Beijing, China; 3 State Key Laboratory of Toxicology and Medical Countermeasures, Beijing Institute of Pharmacology and Toxicology, 100850 Beijing, China; 4 Department of Molecular and Cellular Physiology, Graduate School of Medicine, Kyoto University, Yoshida-konoe-cho, Sakyo-ku, Kyoto, 606-8501 Japan; 5 School of Basic Medical Sciences, Fudan University, 200032 Shanghai, China

## Abstract

Faithful chromosome segregation requires proper chromosome congression at prometaphase and dynamic maintenance of the aligned chromosomes at metaphase. Chromosome missegregation can result in aneuploidy, birth defects and cancer. The kinetochore-bound KMN network and the kinesin motor CENP-E are critical for kinetochore-microtubule attachment and chromosome stability. The linear ubiquitin chain assembly complex (LUBAC) attaches linear ubiquitin chains to substrates, with well-established roles in immune response. Here, we identify LUBAC as a key player of chromosome alignment during mitosis. LUBAC catalyzes linear ubiquitination of the kinetochore motor CENP-E, which is specifically required for the localization of CENP-E at attached kinetochores, but not unattached ones. KNL1 acts as a receptor of linear ubiquitin chains to anchor CENP-E at attached kinetochores in prometaphase and metaphase. Thus, linear ubiquitination promotes chromosome congression and dynamic chromosome alignment by coupling the dynamic kinetochore microtubule receptor CENP-E to the static one, the KMN network.

## Abstract (web summary)

During cell division, faithful chromosome segregation requires proper chromosome congression and dynamic maintenance of the aligned chromosomes. Here, the authors find that LUBAC promotes dynamic chromosome congression and alignment by targeting kinetochore motor CENP-E to the KMN network.

## Introduction

Chromosome missegregation can cause aneuploidy, which is linked to human birth defect and cancer. Precise alignment of chromosomes at the spindle equator is a key step for chromosome segregation and relies on chromosome congression at prometaphase and subsequent stable chromosome alignment at metaphase^1^. Previous work has suggested that the plus end-directed kinetochore motor CENP-E (312-kD) contributes not only to the congression of pole-proximal chromosomes, but also continues to maintain the alignment of aligned chromosomes^2–6^. During prometaphase, the recruitment mechanism of CENP-E to unattached kinetochores is well known and involves the Bub1-Bub3 and BubR1-Bub3 complexes^7,8^. CENP-E transports pole-proximal chromosomes to the metaphase plate during congression, a process that requires its motor activity^2,6,9^. At prometaphase, KNL1 (kinetochore null protein 1), a component of the KMN network, is a critical platform for the recruitment of kinetochore proteins^10,11^. Phosphorylation of KNL1 at MELT motifs mediated by Mps1 is required for the recruitment of Bub1-Bub3 and BubR1-Bub3 complexes^11–15^, which subsequently recruit CENP-E to unattached kinetochores. However, when cells enter metaphase, microtubule attachment releases Mps1 from kinetochores, leading to KNL1 dephosphorylation and reduced levels of Bub1-Bub3 and BubR1-Bub3 complexes from kinetochores on attached chromosomes^16^. On the other hand, CENP-E is still present at these attached kinetochores on aligned chromosomes^3^. This pool of CENP-E maintains the dynamic chromosome alignment at metaphase^2,3^. The mechanism by which CENP-E remains on attached chromosomes is not understood.

Ubiquitin-mediated degradation of critical regulators drives multiple cell cycle transitions. Polyubiquitination of Cyclin B1 and Securin by the anaphase-promoting complex/cyclosome (APC/C) is critical for the metaphase-anaphase transition^17,18^. Ubiquitin chains can be assembled through one of seven lysines (branched ubiquitination) or the N terminus of ubiquitin (linear ubiquitination)^19,20^. Compared to branched ubiquitination, the functions of linear ubiquitination are less well studied. The linear ubiquitin chain assembly complex (LUBAC) complex is the only known E3 that specifically conjugates linear ubiquitin chains to substrates^21–24^. It is mainly implicated in innate immune response^21,25–29^. NEMO is one of the well-characterized substrates of LUBAC in this process^21^. Linear ubiquitination of NEMO by LUBAC does not cause its degradation but rather mediates its oligomerization and subsequent NF-κB activation^21,30,31^.

In this study, we report an unexpected function for LUBAC-dependent linear ubiquitination in chromosome alignment and segregation. LUBAC catalyzes the linear ubiquitination of the kinesin motor CENP-E to specifically anchor it at attached kinetochores on mono-oriented or bi-oriented chromosomes, thus promoting full chromosome congression, dynamic chromosome alignment and accurate chromosome segregation. Furthermore, we demonstrate that KNL1 acts as a receptor of linear ubiquitin chains to anchor CENP-E at attached kinetochores. Thus, linear ubiquitin chains constitute a critical mechanism for chromosome congression and alignment by coupling the dynamic kinetochore microtubule motor CENP-E to the static one, the KMN network. Overall, we reveal the role of linear ubiquitin chains in mitosis, and establish this non-degradation ubiquitination system as a safeguard of chromosome segregation.

## Results

### Targeted siRNA screen identified LUBAC as mitotic regulator

**Fig. 1:**
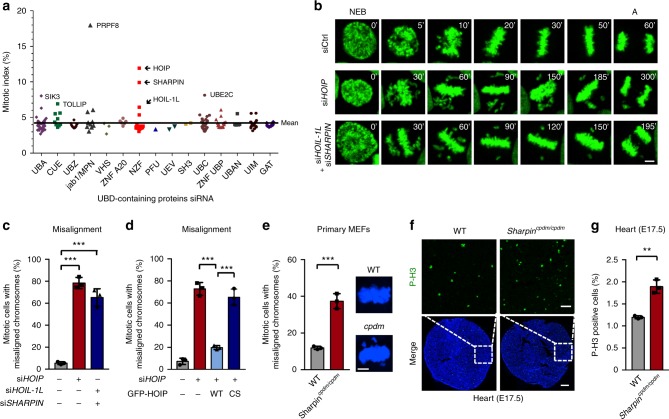
The linear ubiquitin ligase complex LUBAC is required for mitotic progression. **a** The mitotic index of HeLa cells transfected with an arrayed siRNA library targeting 190 genes encoding UBD-containing proteins. Full line, the mean value (4.2%). UBD, ubiquitin-binding domain. Proteins containing two or more UBDs were displayed once. **b** Time-lapse images of control or LUBAC depleted HeLa/GFP-H2B cells. The numbers are minutes after NEB. NEB nuclear envelope breakdown; A anaphase. Scale bar, 5 μm. **c** The percentage of mitotic cells with misaligned chromosomes in **b**. sicontrol, *n* *=* 349 cells; si*HOIP*, *n* *=* 340 cells; si*HOIL-1L* *+* si*SHARPIN*, *n* *=* 377 cells. **d** Expression of GFP-HOIP wild type (WT), but not its ligase-dead mutant (CS), rescued chromosome misalignment in HOIP depleted HeLa/RFP-H2B cells. sicontrol, *n* *=* 118 cells; si*HOIP*, *n* *=* 181 cells; HOIP WT + si*HOIP*, *n* *=* 175 cells; HOIP CS + si*HOIP*, *n* *=* 175 cells. **e** The percentage of mitotic cells with misaligned chromosomes in primary MEF cells from E13.5 embryos of wild-type or *Sharpin*^*cpdm/cpdm*^ mice. WT, *n* *=* 109 cells; *cpdm*, *n* *=* 130 cells. Scale bar, 5 μm. **f** Confocal microscope images of sections of E17.5 heart tissues of wild-type or *Sharpin*^*cpdm/cpdm*^ mice stained with antibody against Pho-H3. Top panels are enlargements of phospho-H3 staining of the regions outlined by white dashed boxes. Green, P-H3; blue, DNA. Scale bars, 200 μm (main image) and 50 μm (magnified region). **g** Quantification of P-H3 positive cells of the entire section in **f**. P-H3-positive cells were calculated automatically by the Volocity 6.0 software. *n* *=* 6 embryos per group. Data are presented as mean ± s.d. of three independent experiments (**c**, **d**, **e** and **g**). **P < 0.01, ***P *<* 0.001; two-sided Student’s *t*-test

Ubiquitination of critical cell-cycle regulators by the E3 ligase APC/C and their orderly degradation is a widely known mechanism that regulates mitotic transitions^17^. We explored the possible involvement of proteins containing ubiquitin-binding domains (UBD) in mitosis, because these proteins control diverse cellular functions by recognizing various types of ubiquitin signals^20^. We transfected HeLa cells with an arrayed small interfering RNA (siRNA) library targeting the genes encoding UBD-containing proteins^20^ (Supplementary Fig. 1a), and used high-content microscopy to screen for proteins required for mitosis by determining the percentage of mitotic cells (mitotic index, MI) (Supplementary Fig. 1b). siRNAs that resulted in the increase by at least 1.5-fold of the average mitotic index (4.2%) were defined as candidate genes. Among them, HOIP (also known as RNF31), HOIL-1L (also known as RBCK1) and SHARPIN (SHANK-associated RH domain interacting protein) (Fig. 1a and Supplementary Fig. 1c, d) were identified as potential mediators of mitosis. These three proteins are the subunits of the linear ubiquitin chain assembly E3 complex LUBAC^32–34^. While LUBAC and M1-linked Ub mainly function in immunity and inflammation, there are limited reports on functions of LUBAC outside of immunity^35,36^. We decided to focus on the possible involvement of this important E3 ligase in mitotic progression.

### The LUBAC complex modulates chromosome alignment

We depleted LUBAC components from HeLa cells stably expressing GFP-tagged histone H2B and monitored their mitotic progression by time-lapse imaging. Because HOIL-1L and SHARPIN are partially redundant adaptors of the catalytic subunit HOIP^33,37^, we co-depleted both in our experiments. Depletion of HOIP or co-depletion of HOIL-1L and SHARPIN caused a prolonged mitotic delay, chromosome missegregation during anaphase and increased mitotic cell death (Fig. 1b and Supplementary Fig. 2a-e). Particularly, LUBAC depletion led to a dramatic increase in the percentage of cells with chromosome misalignment (Fig. 1c and Supplementary Fig. 2f). Similar phenotypes were also observed in U2OS cells (Supplementary Fig. 2g, h). Importantly, the defect of chromosome alignment in HOIP RNAi cells was rescued by the expression of wild-type HOIP, but not its ligase-dead mutant C699S/C702S/C871S/C874S (CS)^19^ (Fig. 1d and Supplementary Fig. 2i). In addition, we observed chromosome misalignment in primary MEF cells from E13.5 embryos of *Sharpin*^*cpdm/cpdm*^ (*cpdm*) mice^32–34^ (Fig. 1e and Supplementary Fig. 2j). We further explored the physiological function of LUBAC by performing pho-H3 staining of heart tissues from E17.5 embryos of *Sharpin*^*cpdm/cpdm*^ (*cpdm*) mice. These tissues indeed showed elevated mitotic index compared to wild-type mice (Fig. 1f, g). Taken together, these data suggest that LUBAC regulates chromosome alignment and mitosis progression under physiological condition, and this mitotic function of LUBAC requires its E3 ligase activity. It has been reported that HOIP-deficient mice die at E10.5 due to TNF-dependent cell death^38^. However, deletion of *Tnf* or *Tnfr1* only partially rescues the lethality of HOIP-deficient mice. Thus, it is likely that the loss of the mitotic function of LUBAC described in our study is a contributing factor to the lethality of HOIP-deficient mice.

### LUBAC affects CENP-E localization at attached kinetochores

**Fig. 2:**
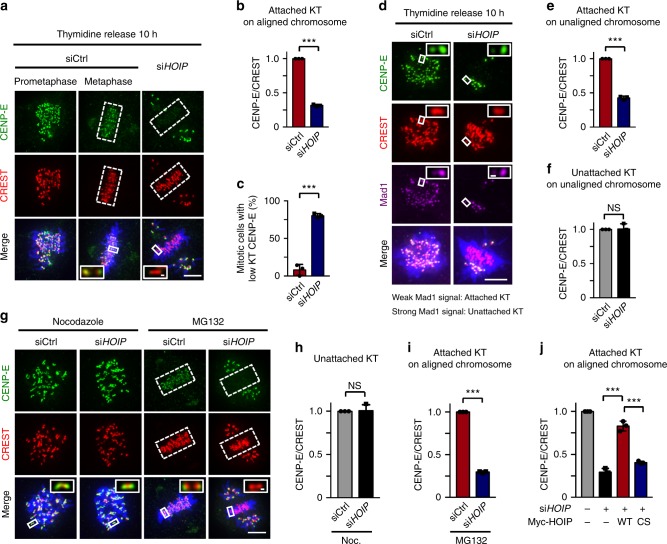
LUBAC is critical for CENP-E localization on attached kinetochores. **a** CENP-E localization in control or HOIP depleted mitotic cells. Green, CENP-E; red, CREST; blue, DNA. **b** Quantification of relative intensity of kinetochore CENP-E on aligned (sicontrol, *n* *=* 23 cells; si*HOIP*, *n* *=* 20 cells) chromosomes in **a**. **c** Percentage of mitotic cells with less than 50% of relative intensity of kinetochore CENP-E on aligned chromosomes of control cells in control (*n* *=* 23) or HOIP depleted (*n* *=* 20) cells. **d** CENP-E localization at unattached (Mad1 signal, strong) kinetochores or attached (Mad1 signal, weak) kinetochores on unaligned chromosomes in control or HOIP depleted mitotic cells. Green, CENP-E; red, CREST; magenta, Mad1; blue, DNA. **e**, **f** Quantification of CENP-E signals at attached kinetochores (**e**) or unattached kinetochores (**f**) on unaligned chromosomes in **d** (sicontrol, *n* *=* 9 cells; si*HOIP*, *n* *=* 12 cells). **g** CENP-E localization in control or HOIP depleted mitotic cells synchronized by Nocodazole or MG132. Green, CENP-E; red, CREST; blue, DNA. **h**, **i** Quantification of relative intensity of CENP-E on unattached (Nocodazole treated) (**h**, *n* *=* 19 cells in each group) or attached (MG132 treated) (**i**, sicontrol, *n* *=* 17 cells; si*HOIP*, *n* *=* 18 cells) kinetochores in **g**. **j** Expression of GFP-HOIP WT, but not CS mutant, rescued CENP-E localization at attached kinetochores on aligned chromosomes. *n* *=* 15 cells in each group. Data are presented as mean ± s.d. of three independent experiments. The average CENP-E/CREST ratio of each group was normalized to control cells. Dashed boxes, regions of aligned chromosomes. Insets, zoomed-in views of the boxed regions. NS not significant, ***P *<* 0.001; two-sided Student’s *t*-test. Scale bars, 5 μm (main image) and 0.3 μm (magnified region)

To investigate how LUBAC regulates chromosome alignment, we next tested the effects of LUBAC knockdown on kinetochore components that are known to play a key role in chromosome alignment. We quantitated the fluorescence intensity of kinetochore components on unaligned and aligned chromosomes in the presence or absence of HOIP. The intensity of the CREST signal was used as a reference for the quantitation to which all fluorescence intensities were normalized^7^. Among the twelve proteins tested, HOIP depletion only affected the kinetochore localization of CENP-E (Supplementary Fig. 3a). The knockdown of HOIP did not alter the expression levels of any of those kinetochore proteins (Supplementary Fig. 3b). Furthermore, we found that knockdown of HOIP specifically affected the kinetochore localization of CENP-E on aligned chromosomes (Fig. 2a, b). Almost 80% of mitotic cells depleted of HOIP exhibited decreased CENP-E intensity on aligned chromosomes (Fig. 2c).

Both sister kinetochores of aligned chromosomes are attached by spindle microtubules (bi-orientation), and kinetochores of unaligned chromosomes might be either attached or unattached by microtubules^39^. We next tested whether LUBAC also affected the localization of CENP-E at attached kinetochores on unaligned sister-chromatids in LUBAC RNAi cells. It is well known that Mad1 is enriched at unattached kinetochores and depleted from kinetochores upon microtubule attachment^16^. Therefore, we used Mad1 localization to indicate the microtubule attachment status of kinetochores on these unaligned chromosomes. LUBAC knockdown indeed specifically decreased the CENP-E intensity at attached kinetochores, but not unattached kinetochores, on these unaligned chromosomes (Fig. 2d-f).

To further confirm this specific effect of LUBAC depletion on CENP-E localization, we examined the kinetochore localization of CENP-E in nocodazole-treated and MG132-treated cells. Nocodazole creates unattached kinetochores by depolymerizing microtubules. MG132 arrests cells at metaphase, with the kinetochores attached to microtubules and aligned at the metaphase plate. Again, HOIP depletion decreased the fluorescence intensity of CENP-E at attached kinetochores in MG132-treated cells, while it did not alter the CENP-E localization at unattached kinetochores in nocodazole-treated cells (Fig. 2g-i and Supplementary Fig. 3c). Similar results were also obtained in HeLa cells co-depleted of the other two subunits of LUBAC (Supplementary Fig. 3d-f). In addition, this effect of LUBAC depletion on CENP-E localization was rescued by wild type HOIP, but not by the ligase dead CS mutant (Fig. 2j and Supplementary Fig. 3g). Furthermore, the localization of Bub1, Bub3, and BubR1 on attached kinetochores was not decreased by LUBAC depletion (Supplementary Fig. 3a). These results suggest that the ligase activity of LUBAC maintains CENP-E at attached kinetochores on chromosomes, independently of known prometaphase kinetochore recruiters of CENP-E.

### LUBAC regulates KT-MT attachment and chromosome alignment

**Fig. 3:**
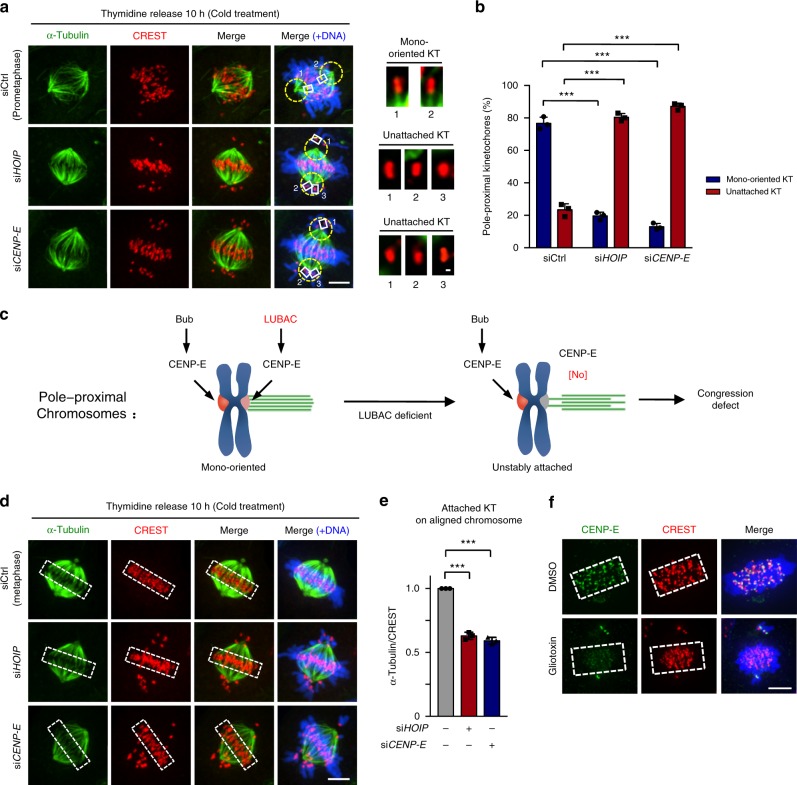
LUBAC regulates KT-MT attachment, chromosome congression and alignment. **a** Cold-stable microtubules at pole-proximal kinetochores in control, HOIP or CENP-E depleted mitotic cells. Green, α-Tubulin; red, CREST; blue, DNA. Yellow circles indicate the pole-proximal region (an area covered by a circle with 2.5 μm in radius centered around the spindle pole). Insets show mono-oriented or unattached pole-proximal kinetochores in each group. **b** Percentage of mono-oriented or unattached pole-proximal kinetochores in control (*n* *=* 29 cells), HOIP (*n* *=* 25 cells) or CENP-E (*n* *=* 25 cells) depleted cells. **c** Model of LUBAC-dependent CENP-E localization at attached kinetochores on pole-proximal chromosomes regulating KT-MT attachment and chromosome congression. **d** Cold-stable microtubules on aligned chromosomes in control, HOIP or CENP-E depleted mitotic cells. Green, α-Tubulin; red, CREST; blue, DNA. White dashed boxes indicate areas where α-tubulin intensity was measured. **e** Relative intensity of cold-stable k-fibers of the attached kinetochores on aligned chromosomes in control, HOIP or CENP-E depleted cells in **d**. *n* *=* 15 cells per group. The average α-tubulin/CREST ratio of each group was normalized to control cells. **f** CENP-E kinetochore localization in HeLa cells treated with 100 nM proteasome inhibitor Velcade for 2 h, and then released into medium containing DMSO or 1.2 μM Gliotoxin (LUBAC inhibitor) for 30 min. Green, CENP-E; red, CREST; blue, DNA. Dashed boxes, regions of aligned chromosomes. Data are presented as mean ± s.d. of three independent experiments (**b** and **e**). Dashed boxes, regions of aligned chromosomes (**d** and **f**). ***P *<* 0.001; two-sided Student’s *t*-test. Scale bars, 5 μm (main image) and 0.3 μm (magnified region)

It is reported that CENP-E contributes to both the congression of pole-proximal chromosomes (in prometaphase) and the maintenance of already aligned chromosomes (in metaphase)^2,3^. In prometaphase, chromosome capture by spindle microtubules is largely a stochastic process. There will be chromosomes with one kinetochore attached and the other unattached (mono-oriented) at this stage^39^. Given that CENP-E is required for kinetochore-microtubule (KT-MT) attachment^4,40^, and LUBAC knockdown affects the CENP-E localization at attached kinetochores on unaligned pole-proximal chromosomes, we next investigated whether these pole-proximal chromosomes became unstably attached when LUBAC was knocked down because of the loss of CENP-E. To test this possibility, we directly observed the microtubule attachment status at kinetochores by cold treatment assay in LUBAC or CENP-E RNAi cells. Similar to the knockdown of CENP-E, depletion of LUBAC also led to polar chromosome misalignment. Interestingly, we found that most pole-proximal mono-oriented chromosomes became unattached when LUBAC or CENP-E was knocked down, indicating that microtubule attachment of these chromosomes is unstable in LUBAC or CENP-E depleted cells (Fig. 3a, b). Taken together, LUBAC knockdown affects the CENP-E localization at attached kinetochores on unaligned pole-proximal chromosomes. Subsequently, these pole-proximal chromosomes become unstably attached to microtubules and thus cannot congress to spindle equator. This finding can explain why pole-proximal chromosomes fail to congress in cells depleted for LUBAC (Fig. 3c).

In metaphase, CENP-E is essential for the stable attachment of kinetochores to microtubule plus ends and for the development of tension across kinetochores on aligned chromosomes^3,4^. Because knockdown of HOIP affected the kinetochore localization of CENP-E on aligned chromosomes (bi-oriented chromosomes), we examined and quantified kinetochore–microtubule fiber (k-fiber) fluorescence (cold treatment) and microtubule-induced tension across sister kinetochores of aligned chromosomes in LUBAC-depleted cells. LUBAC depletion resulted in a decrease of cold-stable k-fibers (Fig. 3d, e) and of average distances between sister kinetochores on aligned chromosomes (Supplementary Fig. 4a, b). These results further suggest that deficient kinetochore association of CENP-E induced by LUBAC depletion reduces the stability of microtubule attachment and kinetochore tension on aligned chromosomes. Thus, although the majority of chromosomes can establish alignment at the spindle equator in CENP-E-deficient or LUBAC-deficient cells, sister kinetochores on these aligned chromosomes cannot maintain stable microtubule attachment and develop normal tension.

CENP-E at attached kinetochores on aligned chromosomes contributes to the maintenance of already aligned chromosomes at metaphase. It is reported previously that inhibition of the motor activity of CENP-E by GSK-923295^41^ leads to a marked depletion of CENP-E from kinetochores on aligned chromosomes and causes chromosome misalignment^3^. We next examined whether LUBAC is required for the maintenance of chromosome alignment. To do so, we treated Velcade-arrested metaphase HeLa cells with the fungal metabolite gliotoxin, which inhibits LUBAC by binding to the RING-IBR-RING domain of HOIP^42^. In non-treated cells, CENP-E localized at the kinetochores on aligned chromosomes (Fig. 3f and Supplementary Fig. 4c). Treating metaphase cells with LUBAC inhibitor Gliotoxin markedly decreased the kinetochore-localized CENP-E on aligned chromosomes (Fig. 3f and Supplementary Fig. 4c). Meanwhile, Gliotoxin hardly induced apoptosis under this condition^43^ (Supplementary Fig. 4d and 4e).

To exclude the possibility that Gliotoxin regulates CENP-E localization through inhibiting or affecting other reported processes and targets^43^, we next detected CENP-E localization at metaphase cells treated with inhibitors targeting the proteasome (MG132), farnesyl transferase^44^ (FTI-277), GGTase^44^ (GGTI 298), or histone methyltransferases^45^ (Chaetocin, G9a and SUV39H1 inhibitor). Our data showed that none of them had an effect on the localization of CENP-E at metaphase cells (Supplementary Fig. 4f, g). Taken together, these results suggested that Gliotoxin regulates CENP-E localization at metaphase most likely by inhibiting the LUBAC E3 ligase activity, but not through targeting other known targets. Of course, we cannot exclude the possibility of the pan-inhibition of multiple known or unknown targets by Gliotoxin as a contributing factor to the observed phenotypes.

High spatial and temporal resolution time-lapse imaging showed that some chromosomes failed to congress to the spindle equator and some of the already aligned chromosomes were not maintained at the metaphase plate in LUBAC knockdown cells (Supplementary Fig. 4h). Collectively, our results so far have demonstrated that LUBAC promotes both the congression of pole-proximal chromosomes and the maintenance of already aligned chromosomes by regulating the localization of CENP-E at attached kinetochores and KT-MT attachment. Our model suggested that LUBAC-dependent CENP-E localization to attached kinetochores of mono-oriented chromosomes promotes congression, and such localization to bi-oriented chromosomes stabilizes chromosome alignment. Therefore, the unaligned chromosomes in LUBAC knockdown cells could be divided into two groups: (I) chromosomes failed to congress and (II) chromosomes initially reached the spindle equator but then exited.

### The kinesin motor CENP-E is a LUBAC substrate in mitosis

**Fig. 4:**
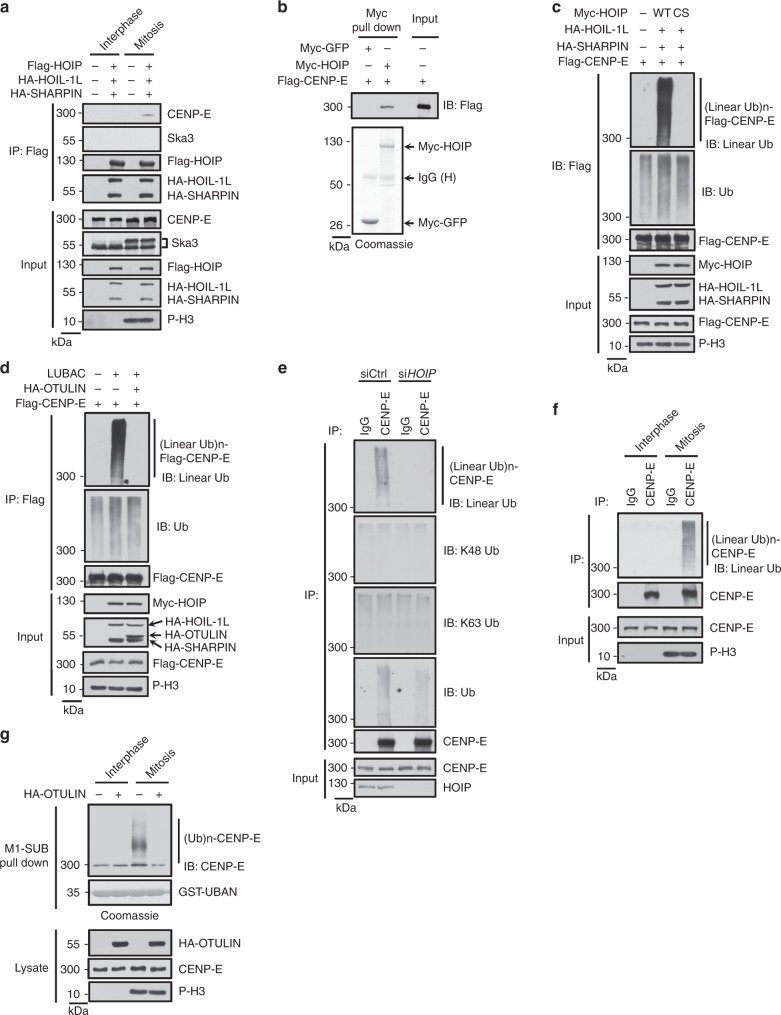
LUBAC attaches linear ubiquitin chains to CENP-E in mitosis. **a** Endogenous CENP-E interacts with ectopic LUBAC in mitotic HeLa cells transfected with LUBAC components. **b** CENP-E binds to HOIP in vitro. Flag-CENP-E protein was purified from sonicated mitotic HEK293T cell lysate by anti-Flag M2 affinity gel and eluted with 3 × Flag peptide, then incubated with Myc-GFP or Myc-HOIP bound beads (pulled down from sonicated mitotic HEK293T cell lysates by anti-c-Myc affinity gel). Top: Proteins retained on the anti-c-Myc affinity gel were then analyzed by western blotting with anti-Flag antibody. Bottom: Purified Myc-GFP or Myc-HOIP were separated by electrophoresis and stained with Coomassie brilliant blue. **c**, **d** HEK293T cells were transfected with Flag-CENP-E, LUBAC WT, CS mutant (**c**) or co-transfected with OTULIN (**d**) as indicated and synchronized into mitosis. Cell lysed as described in Methods section with heating procedure and lysates were immunoprecipitated with anti-Flag M2 affinity gel. The immunoprecipitates and cell lysates were analyzed by immunoblot with indicated antibodies. **e** Linear, K48, K63 or total ubiquitination of endogenous CENP-E was detected in control and HOIP-knockdown HeLa cells synchronized into mitosis. Equal amount of samples were loaded to different gels and were blotted with anti-linear Ub, K48 Ub, K63 Ub or ubiquitin antibody, respectively. **f** The linear ubiquitination level of endogenous CENP-E in interphase and mitosis. **g** Purification of endogenous linear Ub conjugates with M1-SUB (Met1-linkage-specific Ub binder, based on NEMO’s UBAN region) from interphase and mitotic HeLa cell transfected with OTULIN or not. Purified material and lysates were examined by immunoblotting. Loadings of M1-SUB (GST-UBAN) protein were stained with Coomassie

Since the E3 activity of LUBAC is required for CENP-E localization (Figs. 2j and 3f), we next examined whether CENP-E is a substrate of LUBAC. We found that LUBAC specifically interacted with CENP-E, but not the kinetochore protein Ska3, in mitotic cells (Fig. 4a). Meanwhile, purified HOIP could bind to CENP-E in vitro, indicating that HOIP is responsible for the direct interaction between LUBAC and CENP-E (Fig. 4b). Consistently, we observed that Myc-HOIP localized to kinetochores and spindle poles in mitotic cells (Supplementary Fig. 5a). These results suggested that E3 ligase LUBAC could interact with CENP-E in mitosis.

Next, we detected whether CENP-E could be linearly ubiquitinated by LUBAC in mitosis. The result showed that CENP-E was ubiquitinated in mitosis by LUBAC wild type, but not the ligase-dead CS mutant (Fig. 4c), as shown by an antibody that specifically recognized linear polyubiquitin chains. We confirmed that this antibody could not recognize K48-linked or K63-linked polyubiquitin chains (Supplementary Fig. 5b). Moreover, we found that linear ubiquitin chains could also colocalize with CENP-E at kinetochores in LUBAC overexpressed metaphase HeLa cells (Supplementary Fig. 5c). In addition, linear ubiquitination of CENP-E was abolished by co-expression of the linear ubiquitin-specific deubiquitinase OTULIN^22,36,46–49^ (Fig. 4d), which is known to specifically disassemble linear polyubiquitin chains. To test whether LUBAC has the same effect on the endogenous CENP-E in mitosis, we examined the linear ubiquitination of endogenous CENP-E in mitotic cells. Importantly, the linear ubiquitination of endogenous CENP-E, but not K48 or K63 ubiquitination, was reduced by HOIP depletion, indicating that the linear ubiquitination of CENP-E requires LUBAC (Fig. 4e). In addition, the blots for total Ub showed that the ubiquitination level of CENP-E was reduced but still detectable when co-transfected with LUBAC ligase-dead CS mutant or together with OTULIN (Fig. 4c, d). Furthermore, the total ubiquitination of endogenous CENP-E was reduced rather than abolished when HOIP was knocked down (Fig. 4e). Thus, these results indicate that other types of Ub chains might be present on CENP-E in mitosis. It will be interesting to explore the function of these Ub linkages in future studies.

Furthermore, we found that linear ubiquitination of endogenous CENP-E only occurs in mitosis, but not in interphase (Fig. 4f). To further confirm the linear ubiquitination of endogenous CENP-E, we performed pull down assay with GST-coupled M1-SUB^22,50^. Consistently, the linearly ubiquitinated CENP-E could only be detected in mitosis, and the smear was reversed by OTULIN co-expression (Fig. 4g). Taken together, these results suggest that CENP-E is a linear ubiquitination substrate of LUBAC in mitosis.

### Linear ubiquitination regulates CENP-E localization

**Fig. 5:**
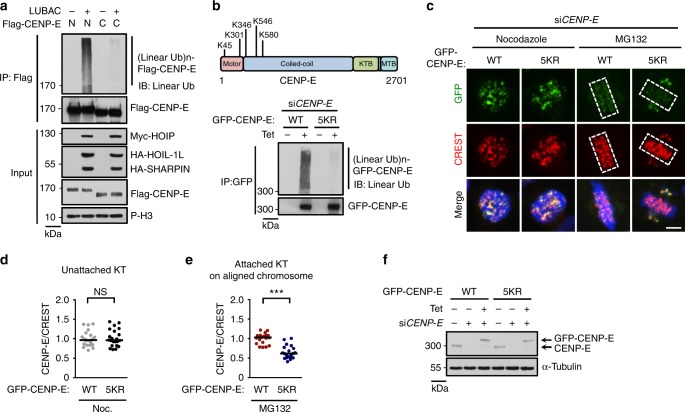
Linear ubiquitination regulates CENP-E localization on attached kinetochores. **a** The linear ubiquitination of CENP-E N-terminal (residues 1-1360) and C-terminal (residues 1340-2701) truncations in mitotic HEK293T cells co-transfected with LUBAC. **b** Top: diagram of CENP-E with identified five major linear ubiquitination sites. K, lysine. Bottom: Flp-In T-REx HeLa stable cell line expressing Tet-inducible GFP-CENP-E WT/5KR mutant were transfected with *CENP-E* siRNA, then were synchronized into mitosis and treated with tetracycline to induce GFP-CENP-E WT or 5KR mutant expression. After 36 h, cells lysates were immunoprecipitated by anti-GFP agarose and the immunoprecipitates were analyzed by immunoblotting. KR mutant: in which identified ubiquitinated lysine (K) sites of CENP-E were substituted with arginines (R). **c** Kinetochore localization of GFP-CENP-E WT or 5KR mutant in Nocodazole (Noc.) or MG132-arrested Flp-In T-REx HeLa stable cell lines depleted of endogenous CENP-E. Green, GFP-CENP-E; red, CREST; blue, DNA. Dashed boxes, regions of aligned chromosomes. Scale bar, 5 μm. **d**, **e** Quantification of GFP-CENP-E signals on unattached (Nocodazole treated) (**d**, *n* = 22 cells in each group) or attached (MG132 treated) (**e**, *n* = 20 cells in each group) kinetochores in **c**. Each dot represents one cell. Bars indicate the median. The average CENP-E/CREST ratio of each group was normalized to control cells. NS not significant, ***P *<* 0.001; two-sided Student’s *t*-test. **f** The knockdown efficiency of endogenous CENP-E and the expression of Tet-inducible GFP-CENP-E WT or 5KR mutant in **c**–**e**

We further identified the LUBAC-catalyzed ubiquitination sites of CENP-E using mass spectrometry^51^. Before doing this, we mapped the interaction region of CENP-E with HOIP, and found that both the N-terminal (residues 1-1360) and C-terminal region (residues 1340-2701) of CENP-E could bind strongly to HOIP (Supplementary Fig. 5d). Moreover, the N-terminal region of CENP-E, but not its C-terminal region, could be efficiently linear-ubiquitinated by LUBAC (Fig. 5a). Thus, we performed the mass spectrometry using the N-terminal region of CENP-E. The ubiquitinated peptides of CENP-E and multiple signature linear ubiquitin peptides containing the sequence GGMQIFVK were identified (Supplementary Data 1, Supplementary Fig. 5e).

We next established Flp-In T-REx HeLa stable cell lines expressing Tet-inducible wild type CENP-E or CENP-E KR mutants. We found that LUBAC-mediated linear ubiquitination of CENP-E 5KR mutant (including K45R, K301R, K346R, K546R, and K580R) was dramatically decreased compared to wild-type CENP-E (Fig. 5b, Supplementary Fig. 5f, g). Moreover, compared to wild type CENP-E, CENP-E 5KR was deficient in localizing to aligned chromosomes although it retained localization on unaligned chromosomes (Fig. 5c-f). Meanwhile, cells expressing CENP-E 5KR mutant were deficient in chromosome alignment (Fig. 5c). Thus, the five lysines, K45, K301, K346, K546, and K580, are the major linear ubiquitination sites of CENP-E required for maintaining chromosome alignment.

### KNL1 is a receptor for linear ubiquitin chains in mitosis

**Fig. 6:**
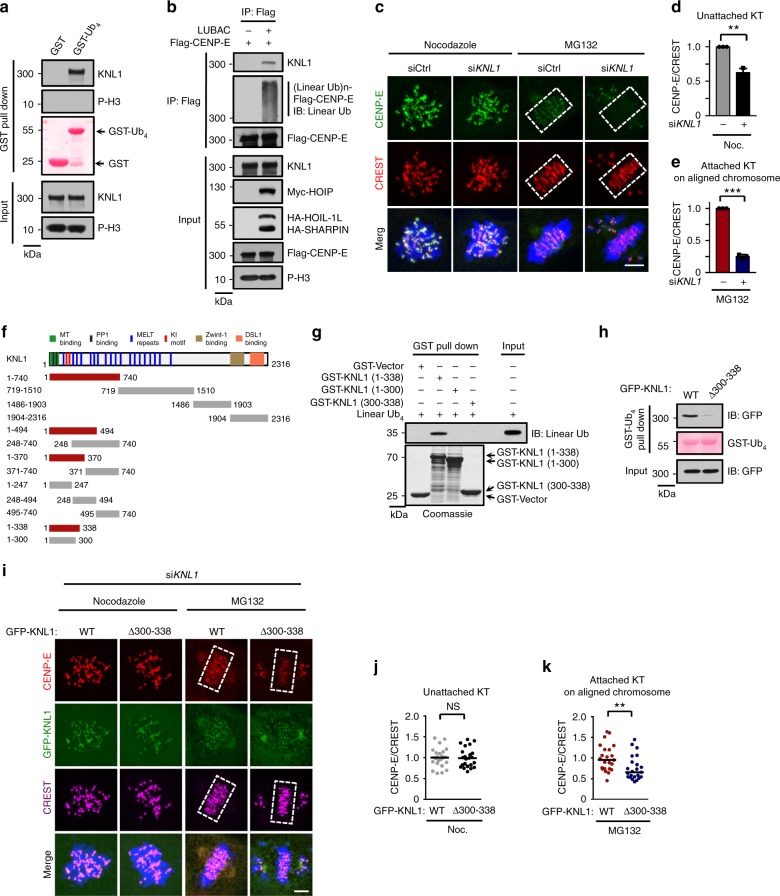
KNL1 is the receptor for linear ubiquitin chains of CENP-E at attached KT. **a** Chromosome isolation fraction of MG132-arrested metaphase HeLa cells was pulled down by GST-tagged linear tetraubiquitin (GST-Ub4), and then endogenous proteins were detected by indicated antibodies. Loadings of GST proteins were determined by Ponceau S staining. **b** Flag-CENP-E was transiently transfected into HeLa cells with or without LUBAC. MG132-arrested metaphase cells were collected, and the immunoprecipitates and cell lysates were analyzed by immunoblot. **c** CENP-E localization in control or KNL1 depleted mitotic cells synchronized by Nocodazole or MG132. Green, CENP-E; red, CREST; blue, DNA. **d**, **e** Quantification of relative CENP-E intensity on unattached (Nocodazole treated) (**d**, *n* = 18 cells in each group) or attached (MG132 treated) (**e**, *n* = 18 cells in each group) kinetochores in **c**. Data are presented as mean ± s.d. of three independent experiments. **f** Schematic diagram showing the regions of KNL1 that interact with the linear ubiquitin chains (linear Ub4). Red line, interaction; gray line, no interaction. **g** The interaction between recombinant GST-fused truncations of KNL1 (residues 1-338) and the linear Ub4 chains. **h** Chromosome isolation fraction of MG132-arrested metaphase Flp-In T-REx HeLa stable cell lines expressing GFP-KNL1 WT or Δ300-338 mutant was pulled down by GST-Ub4, and then GFP-KNL1 was detected by anti-GFP antibody. Loadings of GST proteins were determined by Ponceau S staining. **i** CENP-E localization in Nocodazole or MG132-arrested stable cell lines in **h** depleted of endogenous KNL1. Red, CENP-E; green, GFP-KNL1; magenta, CREST; blue, DNA. **j**, **k** Quantification of CENP-E signals on unattached (Nocodazole-treated) (**j**, GFP-KNL1 WT, *n* = 24 cells; GFP-KNL1 Δ300-338, *n* = 23 cells) or attached (MG132-treated) (**k**, *n* = 23 cells in each group) kinetochores in **i**. Each dot represents one cell. Bars indicate the median. The average CENP-E/CREST ratio of each group was normalized to control cells. Dashed boxes, regions of aligned chromosomes. NS not significant. **P *<* 0.01. ***P *<* 0.001; two-sided Student’s *t*-test in **d**, **e** and **j**, two-sided Mann–Whitney *U* test in **k**. Scale bar, 5 μm

Next, we examined how linear ubiquitin chains of CENP-E regulated its localization on attached kinetochores. In the innate immune response, the linear ubiquitin chains attached to NEMO are recognized by the UBAN domain (ubiquitin binding domain in ABIN and NEMO) within NEMO^30^. We hypothesized that there are receptors for linear ubiquitin chains of CENP-E at attached kinetochores. Based on our bioinformatics analysis, none of the known kinetochore proteins contain a UBAN domain. Next, we enriched possible binding partners of linear ubiquitin chains on mitotic chromosomes using GST-tetraubiquitin and then identified the bound proteins by mass spectrometry (Supplementary Data 2). Interestingly, the kinetochore scaffold KNL1 was among these candidates, and GST-tetraubiquitin could bind to KNL1, but not to pho-H3 (Fig. 6a and Supplementary Fig. 6a).

We decided to test the possible involvement of KNL1 in binding linear ubiquitin chains. We found that only KNL1, but not the Ska complex, purified from mitotic cells could efficiently bind to untagged linear tetraubiquitin chains (Supplementary Fig. 6b). Importantly, the linearly ubiquitinated CENP-E could interact with endogenous KNL1 in MG132-arrested mitotic cells (Fig. 6b). This finding suggests that the linear ubiquitin chains are essential for the CENP-E-KNL1 interaction at metaphase. We next investigated whether the depletion of KNL1, similar to the knockdown of LUBAC, could affect CENP-E localization at attached kinetochores. When KNL1 was knocked down, the localization of CENP-E at unattached kinetochores was expectedly reduced, presumably because KNL1 also recruits CENP-E indirectly through Bub1/BubR1-Bub3 complexes. Importantly, CENP-E localization was affected much more severely at attached kinetochores comparing to unattached ones (Fig. 6c-e), consistent with a role of KNL1 in anchoring ubiquitinated CENP-E at attached kinetochores.

Further mapping experiments showed that the N-terminal region (residues 1-338) of KNL1 was sufficient to bind to the linear ubiquitin chains (Fig. 6f and Supplementary Fig. 6c-e). A slightly smaller region of KNL1 (residues 1-300) could no longer bind to the linear ubiquitin chains (Fig. 6f and Supplementary Fig. 6e). This result suggested that residues 300-338 of KNL1 are critical for binding to the linear ubiquitin chains. This region is unlikely to be sufficient for ubiquitin chain binding, as a KNL1 fragment (residues 248-494) containing this region fails to bind to ubiquitin chains (Fig. 6f and Supplementary Fig. 6d). Importantly, in vitro GST pulldown assay showed that recombinant 1-338 of KNL1, but not 1-300 or 300-388 truncations, was able to bind to the linear ubiquitin chains (Fig. 6g). Taken together, these results indicate that KNL1 might be a direct receptor of linear ubiquitin chains.

The N-terminal region (1-338 aa) of KNL1 (KNL1N) contains several functional motifs, including the microtubule-binding domain (residues 1-68), PP1-binding RVSF and SILK motifs, two KI motifs (KI1 and KI2) and three phospho-MELT motifs phosphorylated by Mps1 that mediate the recruitment of Bub1-Bub3 and BubR1-Bub3 complexes to unattached kinetochores^52,53^. Microtubule attachment leads to PP1 recruitment at attached kinetochores and dephosphorylation of MELT motifs, thus releasing the Bub1-Bub3 and BubR1-Bub3 complexes from KNL1^52^. We performed the linear ubiquitin chains binding assay in vitro by pre-incubating microtubules and PP1 with KNL1N to mimic microtubule attachment status at attached kinetochores. Even in the presence of microtubules and PP1, the KNL1N still efficiently interacted with linear tetraubiquitin chains (Supplementary Fig. 6f). This result suggested that KNL1N might be responsible for interacting with the linear ubiquitin chains of CENP-E at attached kinetochores in vivo.

To further confirm the functional importance of linear ubiquitin binding by KNL1, we created a mutant with its critical ubiquitin-binding region (residues 300-338) deleted and established Flp-In T-REx HeLa stable cell lines expressing Tet-inducible KNL1 WT or the Δ300-338 mutant. Expectedly, KNL1 Δ300-338 mutant was deficient in binding untagged linear ubiquitin chains (Fig. 6h). Strikingly, unlike wild type KNL1, KNL1 Δ300-338 mutant could not restore the fluorescence intensity of CENP-E at attached kinetochores on metaphase chromosomes in MG132-treated KNL1 RNAi cells, while it did not alter the CENP-E localization at unattached kinetochores in nocodazole-treated cells (Fig. 6i-k and Supplementary Fig. 6g). Consequently, cells expressing KNL1 Δ300-338 were deficient in chromosome alignment (Fig. 6i). Importantly, KNL1 Δ300-338 mutant was functional in supporting Bub1 localization at both unattached and attached kinetochores (Supplementary Fig. 6h, i). Therefore, these data suggest a direct mechanism of linear ubiquitination-dependent CENP-E localization at attached kinetochores.

## Discussion

**Fig. 7:**
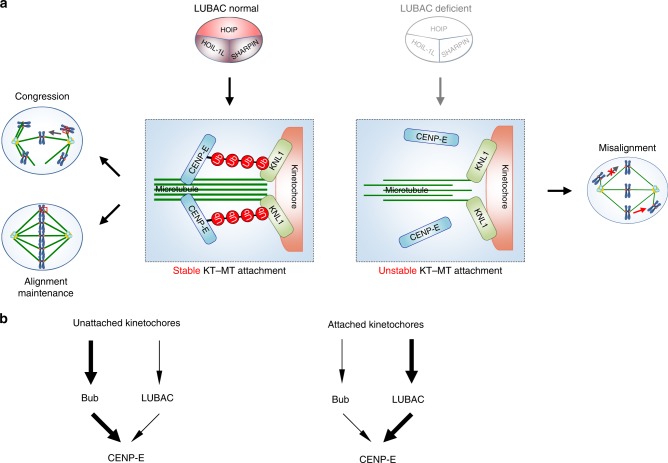
The linear ubiquitin chains of CENP-E anchor it at attached kinetochores. **a** Model of LUBAC controlling chromosome congression and alignment maintenance by mediating the linear ubiquitination of CENP-E. KNL1 then anchors the linear ubiquitin chains on CENP-E at attached kinetochores. **b** At unattached kinetochores, CENP-E is mainly recruited by the Bub1-Bub3 and BubR1-Bub3 complexes (left). Once microtubule attachment releases these recruiters, LUBAC-mediated linear ubiquitination of CENP-E is responsible for anchoring it at attached kinetochores (right)

Faithful chromosome segregation requires proper chromosome congression at prometaphase and maintenance of the aligned chromosomes at metaphase^1,54^. The mitotic motor CENP-E is critical for both the congression of pole-proximal chromosomes and the maintenance of already aligned chromosomes. Our evidence here strongly supports a model in which LUBAC-dependent regulation of CENP-E is critical for establishing (congression) and maintaining dynamic chromosome alignment. In this model, LUBAC mediates the linear ubiquitination of CENP-E and facilitates the anchoring of CENP-E by KNL1 at attached kinetochores. LUBAC-dependent CENP-E localization to attached kinetochores of mono-oriented and bi-oriented chromosomes promotes chromosome congression and alignment through stabilizing kinetochore-microtubule attachment (Fig. 7a).

Chromosome capture by spindle microtubules is a largely stochastic process, and chromosome alignment occurs asynchronously^16,39^. CENP-E is critical for establishing and maintaining chromosome alignment at the spindle equator^2,3^. At unattached kinetochores, CENP-E is mainly recruited by the Bub1-Bub3 and BubR1-Bub3 complexes^7^. Once microtubule attachment releases these recruiters, LUBAC-mediated linear ubiquitination of CENP-E is responsible for anchoring it at attached kinetochores (Fig. 7b). Our discovery provides a mechanism for keeping CENP-E at attached kinetochores. This mechanism is presumably insensitive to microtubule attachment, and thus is fundamentally different from the known microtubule-sensitive CENP-E recruitment mechanisms at unattached kinetochores. Although CENP-E is a major functional substrate of LUBAC during mitosis, we cannot rule out the possible presence of additional target proteins involved in the chromosome alignment process.

We have further defined a direct mechanism by which linear ubiquitin chains promote CENP-E localization and identified KNL1 as a receptor of linear ubiquitin chains at attached kinetochores. The linear ubiquitin chains bind to the N-terminal region of KNL1 in the absence of Mps1 in vitro, indicating that unphosphorylated KNL1 is capable of linear ubiquitin chain binding. The same KNL1 region, when phosphorylated by Mps1, can mediate the recruitment of Bub1-Bub3 and BubR1-Bub3 complexes to unattached kinetochores^53^. One intriguing possibility is that, once microtubule attachment causes KNL1 dephosphorylation and releases the Bub complexes, this KNL1 region is free to bind to the linear ubiquitin chains of CENP-E, keeping CENP-E at attached kinetochores. This might explain why LUBAC only regulates the localization of CENP-E at attached kinetochores, but not at unattached kinetochores. Future experiments are needed to test this hypothesis.

KNL1 depletion from human cells reduces CENP-E localization at both attached and unattached kinetochores, leading to a chromosome misalignment phenotype very similar to that of CENP-E deficiency. Deletion of a critical ubiquitin-binding region of KNL1 recapitulates the chromosome misalignment phenotypes of LUBAC depletion, and only affects CENP-E localization at attached kinetochores. Our functional data establish KNL1 as an important downstream effector of LUBAC-dependent linear ubiquitination. Of course, we cannot exclude the existence of additional receptors of linear ubiquitination in mitosis. In contrast to ubiquitination that leads to degradation, this type of linear ubiquitination strengthens protein-protein interactions for mitotic progression. Our results reveal a non-degradation type of ubiquitin signal for establishing and maintaining chromosomes alignment to ensure subsequent accurate metaphase-anaphase transition^55^.

In conclusion, we have uncovered an unexpected function of LUBAC as a mitotic regulator, and identified KNL1 as a linear ubiquitin chain receptor at attached kinetochores. Chemical inhibitors targeting mitotic regulators, such as Aurora kinases, CENP-E and PLK1, are being developed as anti-cancer drugs^41,56^. Inactivation of LUBAC can cause mitotic cell death. Being an enzyme, LUBAC is a potential mitotic target for anti-cancer therapy. LUBAC inhibitor has been shown to suppress the inflammatory immune response^42,43^. Therefore, targeting LUBAC with small molecules may be a promising approach to treat inflammation-associated cancers.

## Methods

### Mouse strains and animal work

All animal experiments were performed with the approval of the Institutional Animal Care and Use Committee at the National Center of Biomedical Analysis in Beijing. Animals were housed in a pathogen-free facility under a 12-h light cycle. All embryos used for this study were obtained from natural mattings of virgin females of 8–10 weeks of age. Noon on the day of the discovery of a vaginal plug was considered embryonic day E0.5. The SHARPIN-deficient (C57BL/KaLawRij -*Sharpin*^*cpdm*^***/*** RijSunJ, abbreviated to *Sharpin*^*cpdm/cpdm*^) mice were purchased from Jackson Laboratory^57^. *Sharpin*^*cpdm/cpdm*^ mice carry a spontaneous mutation in exon 1 of the *Sharpin* gene, resulting in the frame shift and premature termination of the SHARPIN protein. For genotyping of *cpdm* mice, exon 1 of the *Sharpin* gene was amplified by PCR using primers (Mouse *Sharpin*-F and Mouse *Sharpin*-R, Supplementary Data 3) and analyzed by direct sequencing to identify a guanine deletion. The sex of embryos was not determined for the analyses conducted in this study.

### Cell culture and cell synchronization

HeLa, U2OS and HEK293T cells were obtained from the ATCC. HeLa-GFP H2B stable cell line and HeLa-RFP H2B stable cell line were kindly provided by Dr. Stephen Doxsey and Dr. Chuanmao Zhang. Flp-In T-Rex host HeLa cell line was a kind gift from Dr. Bailong Xiao. To isolate mitotic cells, cells were synchronized by double-thymidine (2 mM, T1895, Sigma) and then released into fresh medium for 10–11 h. To collect prometaphase or metaphase cells in immunofluorescence assays, cells were incubated in thymidine-containing medium for 24 h, further released into fresh medium for 10 h and treated with nocodazole (100 ng/ml, M1404, Sigma) or proteasome inhibitor MG132 (10 μM, 474790, Calbiochem) for 1 h. To collect metaphase cells in linear ubiquitin chains binding assays, cells were synchronized by thymidine–nocodazole arrest and shaken off, then release into MG132 for 2 h.

Primary MEFs were generated from E13.5 embryos in accordance with standard procedures. Second-passage MEFs were used to perform indicated experiments. Cells were grown in Dulbecco’s modified eagle medium (DMEM) with 10% tetracycline-free fetal bovine serum, 100 U/ml penicillin, and 100 U/ml streptomycin in a humidified incubator at 37 °C and 5% CO_2_.

### siRNA Library screening and high-content microscopy

Custom human siRNA library targeting ubiquitin binding domains (UBD) containing proteins was designed and synthesized with a pool of four siRNA duplexes for each gene arrayed in a 96-well format (Dharmacon). In total, the UBDs include 15 types and the UBD-containing proteins siRNA library targets 190 genes. Cells were transfected with siRNAs and stained as described in Supplementary Fig. 1b. Then fluorescence images of stained cells were collected using an IN Cell Analyzer 2000 high content fluorescence microscope (GE Healthcare) and analyzed by the IN Cell Analyzer Workstation 3.7 using Multi Target Analysis Module. For all measurements, 9 fields in each well (>10,000 cells) were collected with a ×20 objective. The Threshold Filter was used to screen positive cells with pSer10-H3 staining intensities over 160 gray level.

### Transfection with siRNA and DNA

siRNAs were performed with Lipofectamine RNAiMAX Transfection Reagent (13778, Invitrogen). The 1# siRNAs targeting three LUBAC components were used in most of the LUBAC depletion experiments in this study unless indicated otherwise (Supplementary Data 3). For the rescue assays in HOIP depleted cells, 12 h before siRNA transfection, RNAi-resistant plasmids were transfected using TurboFect transfection reagent (R0531, Thermo Scientific). Following transfection, cells were synchronized into mitosis and examined by time-lapse imaging or immunostaining. For the experiments involving GFP-CENP-E WT/5KR mutant or GFP-KNL1 WT/Δ 300-338 mutant, CENP-E siRNA or KNL1 siRNA was transfected into Flp-In T-REx HeLa stable cell lines expressing Tet-inducible GFP-CENP-E WT/5KR mutant or GFP-KNL1 WT/Δ 300-338 mutant. After transfection 12 h, cells were synchronized into mitosis as described above in 'Cell culture and cell synchronization' section and added with tetracycline (1 μg/ml, 58346, Calbiochem) to induce GFP-CENP-E WT/5KR mutant or GFP-KNL1 WT/Δ 300-338 mutant expression.

### Constructs

Myc-HOIP WT and HA-HOIL-1L were supplied by Dr. Kazuhiro Iwai. GFP-CENP-E WT was a kind gift from Dr. Don W. Cleveland. HA-OTULIN and GST-M1-SUB was obtained from Dr. Mads Gyrd-Hansen. GST-Ub4 was provided by Dr. Ivan Dikic. Flag-KNL1, Myc-Ska1 and Myc-Ska2 were supplied by Dr. Hongtao Yu. HA-SHARPIN, Flag-CENP-E, GFP-HOIP, Flag-Ska3 or GFP-KNL1 WT/Δ 300-338 mutant, Myc-GFP were amplified by PCR and cloned into pXJ40, pcDNA5/FRT/TO, EGFP-N1 or pcDNA3.0 (Supplementary Data 3). Flag-CENP-E and Flag-KNL1 truncations were amplified from full length CENP-E or KNL1 and cloned in pcDNA3.0. HOIP CS mutant, CENP-E 5KR mutant and all the siRNA-resistant plasmids described here were generated by PCR-based site-directed mutagenesis (Supplementary Data 3).

### Generation of Flp-In T-REx HeLa stable cell lines

To generate Flp-In T-REx HeLa stable cell line expressing Tet-inducible GFP-CENP-E WT/5KR mutant or GFP-KNL1 WT/Δ 300-338 mutant, Flp-In T-REx host HeLa cell line was cotransfected with pOG44 and pcDNA5/FRT/TO (Invitrogen) inserted with a siRNA-resistant ORF of GFP-CENP-E WT/5KR mutant or GFP-KNL1 WT/Δ300-338 mutant, then selected with hygromycin B (250 μg/ml, 10687010, Invitrogen). To induce target gene expression, cells were treated with tetracycline (1 μg/ml) 36 h before analysis.

### Time-lapse imaging

HeLa/GFP-H2B, U2OS/GFP-H2B and HeLa/RFP-H2B stable cell lines were used to performed the time-lapse assays^58,59^. For the high spatial and temporal resolution time-lapse imaging, ten 1.5 μm-separated z-planes covering the entire volume of mitotic cells were collected every 2 min as indicated.

### Analysis of CENP-E linear ubiquitination

Flag-CENP-E was transiently transfected into HEK293T cells with or without Myc-HOIP, HA-HOIL-1L, HA-SHARPIN and ubiquitin, cells were synchronized into mitosis and lysed with RIPA buffer containing 20 mM Tris-HCI pH 7.5, 150 mM NaCl, 10 mM EDTA, 1% Triton X-100, 1% Deoxycholate, complete protease inhibitor (04-693-132-001, Roche), phosSTOP (4906837001, Roche) and N-Ethylmaleimide (E3876, Sigma). Cell lysates were sonicated and centrifuged at 15,000×*g*, SDS was added into the supernatants to a final concentration of 1%, followed by heating at 90 °C for 10 min to remove noncovalent associates. Then the samples were diluted to 0.1% SDS and were immunoprecipitated by anti-Flag M2 affinity gel (A2220, Sigma). The immunoprecipitants were detected by immunoblotting with anti-linear Ub antibody. To detect linear ubiquitination of endogenous CENP-E in HOIP-depleted mitotic cells, HeLa cells were transfected with control or *HOIP* siRNA. After 36 h, cells were synchronized into mitosis, then lysed as described above without heating procedure, cell lysates were immunoprecipitated by rabbit anti-CENP-E polyclonal antibody (sc-22790, Santa Cruz) or normal rabbit IgG antibody (sc-2027, Santa Cruz). To detect linear ubiquitination of CENP-E in Flp-In T-REx HeLa stable cell lines expressing Tet-inducible GFP-CENP-E WT or 5KR mutant, cells were transfected with *CENP-E* siRNA, then were synchronized into mitosis and treated with tetracycline to induce GFP-CENP-E WT or 5KR mutant expression. After 36 h, cells were lysed as described above without heating procedure, cell lysates were immunoprecipitated by anti-GFP agarose (D153-8, MBL). The immunoprecipitants were detected by immunoblotting with anti-linear Ub antibody.

### Isolation of endogenous linear ubiquitin conjugates

For isolation of linear Ub chains modified proteins, recombinant protein containing one copy of the UBAN region from human NEMO (residues 257-346) fused to Glutatione-Stransferase (GST) was used^22^(M1-SUB). The interphase and mitotic HeLa cells transfected with or without HA-OTULIN were lysed with buffer (20 mM Na_2_HPO_4_, 20 mM NaH_2_PO_4_, 1% NP-40, 2 mM EDTA) containing 1 mM DTT, 5 mM N-Ethylmaleimide (Sigma), complete protease inhibitor, phosSTOP. Cell lysates were then sonicated and cleared, mixed with 100 μg/mL GST coupled M1-SUB protein bound to Glutathione Sepharose 4B beads (GE Healthcare) and rotated at 4 °C overnight. Beads were washed four times in ice-cold PBS containing 0.1% Tween-20. The bound materials were detected by immunoblotting with anti-CENP-E antibody.

### Immunofluorescence and antibodies

For kinetochore localization and inter-kinetochore distance experiments, mitotic cells were fixed with 1% paraformaldehyde for 5 min at room temperature. For microtubule-CREST double staining to measure inter-kinetochore distances, or to explore the localization of Myc-HOIP in LUBAC co-transfected mitotic cells, HeLa cells were permeabilized in warmed PHEM buffer (25 mM HEPES at pH 6.9, 10 mM EGTA, 60 mM PIPES and 2 mM MgCl_2_) containing 0.1% Triton X-100 for 30 s at room temperature, followed by incubation with 4% paraformaldehyde for 10 min at 37 °C. For analysis of cold-stable microtubules, cells were incubated for 5 min on ice in L-15 medium (Invitrogen) with 20 mM Hepes at pH 7.3, then fixed for 10 min at room temperature with 3.7% formaldehyde in 100 mM Pipes at pH 6.8, 10 mM EGTA, 1 mM MgCl_2_ and 0.2% Triton X-100 ^60^. To stain anti-linear ubiquitin antibody in mitotic HeLa cells, metaphase chromosome spread was prepared according to Earnshaw W.C. et al.^61^. Briefly, the coverslip was swollen in 75 mM KCl for 20–25 min at room temperature, then spun in a table-top centrifuge at maximum acceleration and stopped till the speed reach 2000–2400×*g*. Rapidly remove the coverslip to 1× PBS-azide solution (PBS containing 1 mM EGTA, 0.01% NaN_3_), followed by incubation with 4% paraformaldehyde for 10 min at room temperature.

Immunostaining was performed with the following antibodies: rabbit anti-phospho histone H3 (Ser 10) (1:200, 05-817R, Millipore), mouse anti-CENP-E (1:200, ab5093, Abcam), mouse anti-Bub1 (1:200, B0561, Sigma), rabbit anti-BubR1 (1:200, A300-386A, BETHYL), rabbit anti-SKA1 (1:200, a gift from Dr. Iain M. Cheeseman), rabbit anti-Aurora B (1:200, A300-431A, BETHYL), mouse anti-Hec1 (1:200, ab3613, Abcam), rabbit anti-Spc25 (1:200, S1215, Epitomics), rabbit anti-MCAK (1:200, AKIN05, cytoskeleton), rabbit anti-KNL1 (1:200, NBP1-42704, Novus), rabbit anti-Mis12 (1:200, sc-98368, Santa Cruz), rabbit anti-Nuf2 (1:200, 15731-1-AP, Proteintech), mouse anti-α-tubulin (1:1000, T5168, Sigma), mouse anti-Myc (1:500, sc-40, Santa Cruz), CREST antisera (1:400, 15-234, Antibodies Incorporated), rabbit anti-Mad1 (1:500, a gift from Dr. Hongtao Yu), rabbit anti-CENP-F (1:200, 20982-1-AP, Proteintech), anti-linear Ub^62^ (1:1000, a gift from Dr. Vishva M. Dixit, Genentech Inc.). Cross-adsorbed secondary antibodies from Jackson lab were used, and DNA was stained with Hoechst 33342 (1:1000, H3570, Invitrogen). The following inhibitors were used to treat HeLa cells before immunostaining: Velcade (100 nM, S1013, Selleckchem), Gliotoxin (1.2 μΜ, ab142437, Abcam), FTI 277 (10 μM, S7465, Selleckchem), GGTI 298 (10 μM, S7466, Selleckchem), Chaetocin (1.3 μΜ, S8068, Selleckchem). GFP-Bub3 stable cell line (a gift from Dr. Xueliang Zhu) was used to determine the relative intensity of Bub3. To distinguish unattached and attached kinetochores on unaligned chromosomes, we used Mad1 localization as the indicator, kinetochores with strong Mad1 signal are unattached ones and kinetochores with weak Mad1 signal are attached ones.

Images were acquired with a ×63/1.40 or ×100/1.40 Oil objective on Nikon Eclipse Ti-E Microscope with an UltraView spinning-disc confocal scanner unit (Perkin Elmer) (Figs. 3 and 5), a ×63/1.40 Oil objective on Zeiss LSM 880 system (Fig. 6) or a ×100/1.40 Oil objective on a DeltaVision Image Restoration Microscope (Applied Precision Instruments) (Figs. 2 and 3). All acquisition settings were kept constant for experimental and control groups in the same experiment. The representative images acquired by DV system were processed by iterative constrained deconvolution (SoftWoRx, Applied Precision Instruments). Maximal intensity projections of the entire Z-stack are shown, and optical sections show individual kinetochores more clearly (insets).

To count mono-oriented or unattached pole-proximal kinetochores in cold treatment assay, CREST staining was used to identify kinetochore pairs in individual Z-stacks. We defined the pole-proximal region as an area covered by a circle with 2.5 μm in radius centered around the spindle pole. Each kinetochore was characterized as attached or unattached, depending on whether microtubule fibers ended at the kinetochore. Kinetochores for which the attachment state could not be clearly determined were not counted.

All quantification was performed on raw images without deconvolution. Briefly, the raw images were projected and exported as tiff files, and further analyzed with using ImageJ (National Institutes of Health) software. To quantify the relative intensity of kinetochore components, kinetochore regions were selected based on CREST signal, and an area with the same size was selected in non-kinetochore regions for background determination. The relative kinetochore intensity of a given protein was calculated as the mean of its background-subtracted kinetochore intensity divided by the background-subtracted kinetochore intensity of CREST. To quantify the relative intensities of k-fibers, microtubule signals were quantified by measuring the total fluorescent intensity of a 60 × 200 pixel area on the spindle equator, and normalizing to the signals of CREST with Image J. A 10 × 10 pixel area was selected in non-tubulin regions for background determination.

### Immunohistochemistry

For immunofluorescence staining of heart tissues, tissue samples from mice were frozen in Tissue-Tek O.C.T compound. Sections (6 μm) were cut and fixed in 4% paraformaldehyde, then blocked overnight with 3% normal goat serum. Sections were stained with rabbit anti-phospho Ser10 histone H3 (P-H3). Nuclei were visualized using Hoechst. Images were acquired with a ×10/0.45 objective on Zeiss LSM 880 system and analyzed by the Volocity 6.0 software.

### Western blot

Antibodies used for western blot were: mouse anti-Flag (1:5,000, F3165, Sigma), mouse anti-Myc (1:1,000 sc-40, Santa Cruz), mouse anti-HA (1:1,000, sc-7392, Santa Cruz), mouse anti-GFP (1:500, sc-9996, Santa Cruz), mouse anti-α-tubulin (1:1,000, T5168, Sigma), rabbit anti-phospho-histone H3 (Ser 10) (1:5,000, 05-817 R, Millipore), rabbit anti-BubR1 (1:5000, A300-386A, BETHYL), mouse anti-Bub1 (1:1000, B0561, Sigma), rabbit anti-Bub3 (1:1000, ab131157, Abcam), rabbit anti-CENP-E (1:5,000, a gift from Dr. Don W. Cleveland), sheep anti-HOIP and anti-HOIL-1L (1:5,000, a gift from Dr. Philip Cohen), rabbit anti-SHARPIN (1:1,000, #4444, CST), anti-linear Ub^62^ (1:2500, a gift from Dr. Vishva M. Dixit, Genentech Inc.), rabbit anti-Ub K48 (1:1000, 05-1307, Millipore), rabbit anti-Ub K63 (1:1000, 05-1308, Millipore), rabbit anti-Ska3 (1:3000, a gift from Dr. Hongtao Yu), rabbit anti-KNL1 (1:5000, a gift from Dr. Hongtao Yu). Western blot antibody against SHARPIN of primary MEF cells was: rabbit anti-SHARPIN (1:500, Millipore). Images have been cropped for presentation (uncropped scans of the blots were shown in Supplementary Fig. 7).

### Chromosome isolation

Mitotic cells were collected and lysed with lysis buffer containing 25 mM Tris pH 7.5, 10 mM NaCl, 5 mM MgCl_2_, 0.1% NP40, 10% Glycerol, 5 mM NaF, 10 mM beta-Glycerophosphate, 0.3 mM Na_3_VO_4_, complete protease inhibitor, phosSTOP and 1 mM DTT (lysis buffer: cell pellet = 3:1). Cells were suspended with syringe for 5–10 times, incubated on ice for 5–10 min, and then spun at 3000 g. Discard the supernatant without disturbing the pellet (this portion is the chromosome isolation fraction). Wash the pellet gently with lysis buffer for 3–5 times, then resuspend it with RIPA buffer. The lysate was sonicated and centrifuged at 15,000 × *g*. The supernatant portion was used in the GST-Ub4 pull-down assay.

### Mass spectrometric analysis

To isolate ubiquitinated CENP-E for MS analysis, HEK293T cells were transiently transfected with Flag-CENP-E (1-1360 aa), LUBAC and ubiquitin, then cells were synchronized into mitosis and then lysed as described above in 'Analysis of CENP-E linear ubiquitination' section. Cell lysates were immunoprecipitated by anti-Flag M2 affinity gel, and then Flag-CENP-E immunoprecipitants were eluted with 3× Flag peptide (F4799, Sigma-Aldrich) in elution buffer (50 mM Tris-HCl, pH 7.4, 150 mM NaCl) for 2 h. The eluted samples were concentrated and buffer exchanged into 6 M urea with ultra-centrifugal filter (Sartorius, 50k filter), and the sample were transferred into a PCR tube, sequentially reduced with 10 mM DTT and alkylated with 20 mM Chloroacetamide. Then the sample mixture were added to the ultra-centrifugal filter (Sartorius, 10k filter), concentrated and buffer exchanged into 50 mM NH_4_HCO_3_, and the concentrate were subjected to proteolytic digestion by incubating with 15 ng/μL trypsin overnight at 37 °C in the filter. The tryptic peptides were collected as a filtrate. The remaining sample in the filter were added with 10 ng/μL trypsin again and incubated at 37 °C for another 2 h. All of the tryptic peptides were collected and lyophilized for MS analysis. Peptides were resuspended with 0.1% formic acid, and analyzed by an ultra-performance LC-MS/MS platform of Q Exactive HF mass spectrometer equipped with an easy-nLC1200 liquid chromatography system (Thermo Fisher Scientific, San Jose, CA). To identify GST-Ub4 binding proteins, chromosome isolation fraction of MG132-arrested metaphase HeLa cells was pulled down by GST-Ub4, protein samples were denatured and separated by SDS-PAGE gel then stained with Bio-Safe Coomassie (Bio-Rad). Targeted bands were cut down and digested with trypsin. LC-MS/MS analyses were performed on an Easy-nLC 1000 liquid chromatography system (Thermo) coupled to a LTQ-Orbitrap Fusion (Thermo) via a nano-electrospray ion source.

For raw MS file from CENP-E ubiquitylation, raw file was searched against the human National Center for Biotechnology Information (NCBI) Refseq protein database in Proteome Discoverer 2.1 suited with Mascot software (version 2.3.1, Matrix Science) to achieve a false discovery rate of <1%. The mass tolerance was set to be 20 ppm for precursor, and it was set 50 mmu for the tolerance of product ions. Oxidation (M), Acetyl (Protein-N-term), GlyGly (K) were chosen as variable modifications and Carbamidomethyl (C) was set as fixed modification, and up to two missed cleavages were allowed for trypsin digestion^63^. For raw MS file from GST-Ub4 binding proteins, raw file was searched against the human National Center for Biotechnology Information (NCBI) Refseq protein database in Proteome Discoverer 1.4 suited with Mascot software (version 2.3.1, Matrix Science) to achieve a false discovery rate of <1%. The mass tolerance was set to be 20 ppm for precursor, and it was set 0.5 Da for the tolerance of product ions. Oxidation (M), Acetyl (Protein-N-term), Phospho (Y), Phospho (ST), and DeStreak (C) were chosen as variable modifications; and up to two missed cleavages were allowed for trypsin digestion.

Protein identification data (accession numbers, peptides sequence, sequence coverage etc.) are available in Supplementary Data 1 (Mass spectrometry data for identifying CENP-E ubiquitination sites) and Supplementary Data 2 (Mass spectrometry data for identifying GST-Ub4 interacting proteins).

### Linear ubiquitin chains binding assay

GST and GST-Ub4 proteins were expressed in *Escherichia coli* and were purified by glutathione Sepharose 4B chromatography according to the manufacturer’s protocol (Amersham Biosciences). For GST-Ub4 pull-down assay in vitro, all plasmids of Flag-KNL1 truncations were translated in vitro with a TNT T7 Quick Coupled Transcription/Translation Systems (Promega). Then the system was diluted in PDB buffer (150 mM NaCl, 50 mM Tris -HCl pH 7.5, 5 M DTT and 0.1% NP‐40) and incubated with GST-Ub4 protein. For binding assay between Flag-tagged proteins and untagged linear Ub4 experiments, Flag-KNL1 (wild type), Flag-NEMO (wild type), Flag-NEMO (DF/NA, ER/AA) mutant or Flag-Ska3/Myc-Ska1/Myc-Ska2 was transfected into HEK293T cells, and then extracts from MG312-arrest metaphase 293T cells were prepared. Immunoprecipitation was carried out with mouse anti-Flag M2 affinity gel. These immunoprecipitated Flag-tagged proteins were incubated together with linear Ub4 (Boston Biochem company) overnight at 4 °C in PDB buffer supplemented with 0.5 mg/ml BSA, then washed five times with PDB buffer.

### Statistical analysis

Statistical comparisons between two groups were carried out by two-sided Student’s *t*-test or the two-sided Mann–Whitney *U* test when a normal distribution could not be assumed. Statistical calculations were performed with SPSS software. For all tests, we tested data for normality and variance, and differences were considered statistically significant if *P* values were less than 0.05 (as indicated with *, *P < 0.05; **P < 0.01; ***P < 0.001; NS not significant). No statistical methods were used to predetermine sample size. The experiments were not randomized. No samples were excluded. The investigators were blinded to assess all the staining and time-lapse assays.

### Reporting summary

Further information on experimental design is available in the Nature Research Reporting Summary linked to this article.

## Supplementary information

Supplementary Information

Peer Review File

Description of Additional Supplementary Files

Supplementary Data 1

Supplementary Data2

Supplementary Data 3

Reporting Summary

## Data Availability

All mass spectrometry proteomics data have been deposited to the ProteomeXchange Consortium via the PRIDE partner repository with the dataset identifier PXD011786. The authors declare that all data supporting the findings of this study are available within the article and its Supplementary Information files, or from the corresponding authors upon reasonable request.
