# Supplementary material for: LUBAC controls chromosome alignment by targeting CENP-E to attached kinetochores
Source: Nat Commun. 2019 Jan 17;10:273. doi: 10.1038/s41467-018-08043-7 (PMC6336796; doi:10.1038/s41467-018-08043-7)
Supplement: Supplementary file 1 — Supplementary Information [file 41467_2018_8043_MOESM1_ESM.pdf]

LUBAC controls chromosome alignment by targeting CENP-E to attached kinetochores

Wu *et al.*

Supplementary Information

a

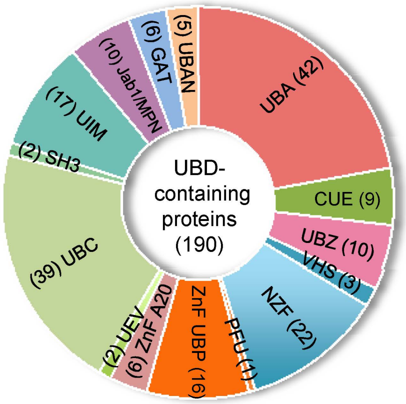

b

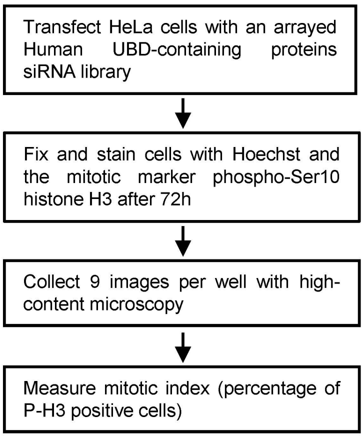

c

	Gene Symbol	Ubiquitin-binding domain	Gene ID	Mitotic Index (%)
Candidate genes	PRPF8	Jab1/MPN	10594	17.987
	HOIP	UBA、NZF	55072	11.941
	SHARPIN	NZF	81858	9.909
	UBE2C	UBC	11065	8.142
	SIK3	UBA	23387	8.037
	TOLLIP	CUE	54472	6.913
	HOIL-1L	NZF	10616	6.445

d

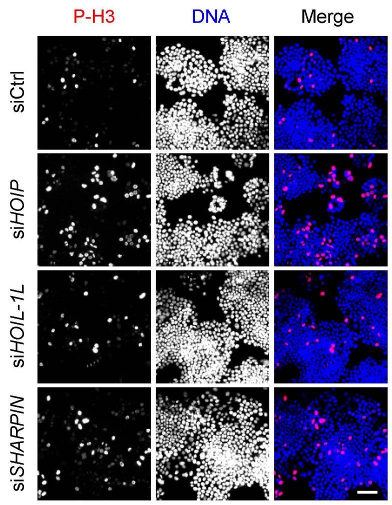

Supplementary Fig. 1 Human UBD-containing proteins siRNA library screen. a Graphical depiction of 190 UBD-containing proteins (15 UBDs) targeted by the siRNA library. Proteins containing two or more UBDs were displayed once. **b** Schematic of high-content microscopy screening protocol. **c** Table of candidate genes from siRNA library screening. **d** Images from high-content microscopy screening in control RNAi or LUBAC components RNAi cells. Red, pho-H3; blue, DNA. Scale bar, 100 μm.

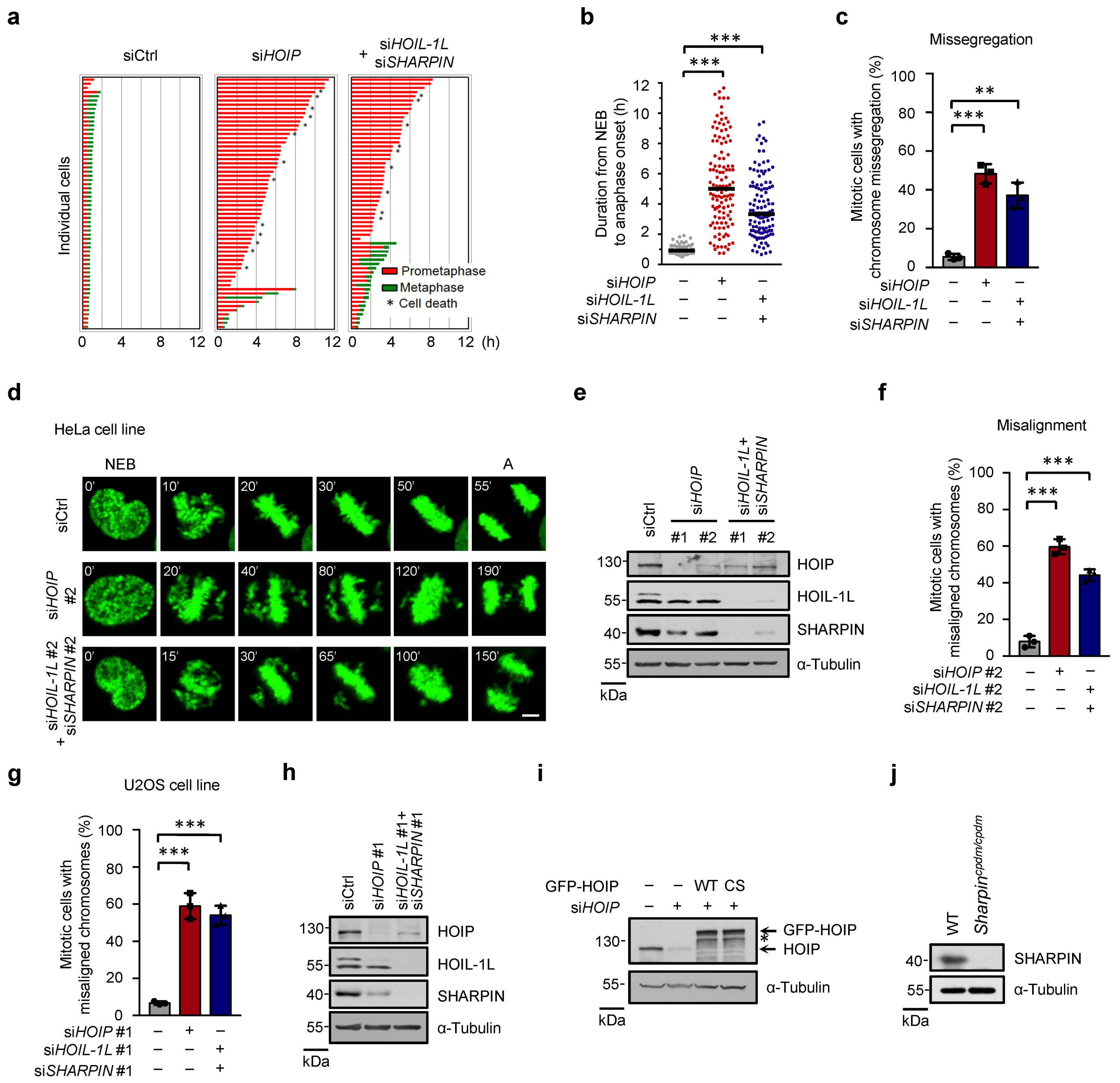

Supplementary Fig. 2 LUBAC is required for mitotic progression. a Mitotic progression of 60 cells per group observed in Fig. 1b. Red bars, prometaphase; green bars, metaphase; bars without asterisk，entry into anaphase； bars with asterisk, cell death preceded by chromosome misalignment. **b** Duration time from NEB to anaphase onset of each cell in Fig. 1b. *n* = 120 cells each group. Bars indicate the median. **c** The percentage of mitotic cells with chromosome missegregation in Fig. 1b. sicontrol, *n* = 349; si*HOIP*, *n* = 340; si*HOIL-1L*+si*SHARPIN*, *n* = 377. **d** Time-lapse images of HeLa/GFP-H2B cells transfected with control, *HOIP* #2 or co-transfected *HOIL-1L* #2 and *SHARPIN* #2 siRNAs. The numbers are minutes after

NEB. NEB, nuclear envelope breakdown; A, anaphase. Scale bar, 5 μm. **e** Knockdown efficiency of siRNAs targeting the indicated proteins in (**a**-**d**) and Fig. 1b and 1c. **f** The percentage of mitotic cells with misaligned chromosomes in **d**. sicontrol, n = 334; si*HOIP* #2, n = 452; si*HOIL-1L* #2+si*SHARPIN* #2, n = 438. **g**, **h** The percentage of mitotic cells with misaligned chromosomes (**g**) or knockdown efficiency (**h**) of control or LUBAC depleted U2OS/GFP-H2B cells. sicontrol, n = 193; si*HOIP* #1, n = 151; si*HOIL-1L* #1+si*SHARPIN* #1, n = 159. **i** The knockdown efficiency of endogenous HOIP and the expression of HOIP siRNA-resistant GFP-HOIP WT or CS mutant in Fig. 1d. '*' indicates an unspecific band. **j** Immunoblots of primary MEF cell lysates in Fig. 1e with the indicated antibodies. Data are presented as mean ± s.d. of three independent experiments (**c**, **f** and **g**). **P < 0.01. ***P < 0.001; two-sided Student's t-test in **c**, **f** and **g,** two-sided Mann-Whitney U test in **b**.

a

Protein	Protein intensity on KT without HOIP	
	Unaligned	Aligned
Ska1	1.04 ± 0.21	1.07 ± 0.20
Bub1	0.97 ± 0.09	0.91 ± 0.21
BubR1	1.14 ± 0.12	0.91 ± 0.13
Bub3	1.06 ± 0.19	1.02 ± 0.15
CENP-E	0.96 ± 0.06	0.29 ± 0.13
Aurora B	0.94 ± 0.19	0.90 ± 0.22
Hec1	1.02 ± 0.13	0.91 ± 0.03
Spc25	0.97 ± 0.18	0.98 ± 0.12
MCAK	1.08 ± 0.07	1.03 ± 0.11
KNL1	1.00 ± 0.18	1.06 ± 0.17
Mis12	0.99 ± 0.12	0.91 ± 0.07
Nuf2	0.98 ± 0.15	1.00 ± 0.16
CENP-F	0.95 ± 0.10	0.83 ± 0.16

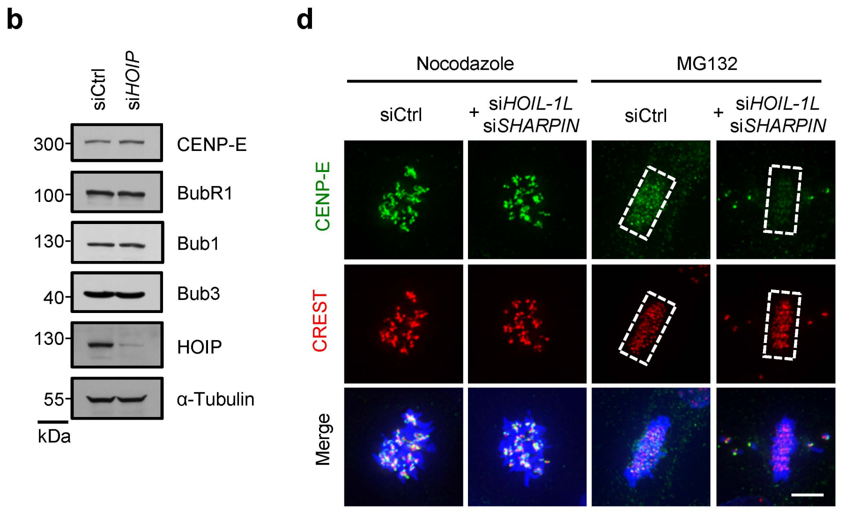

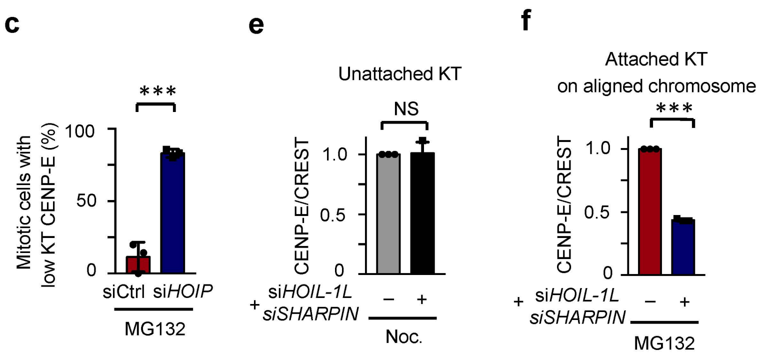

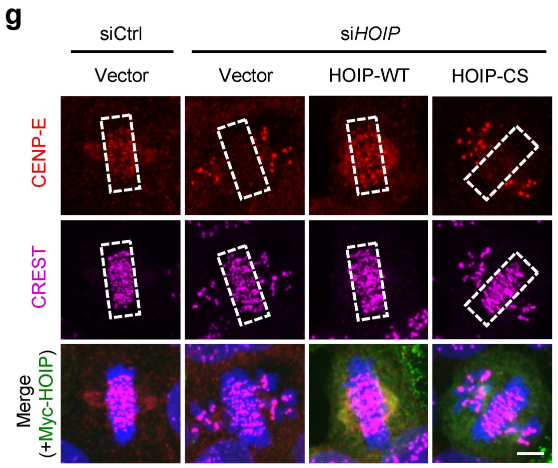

Supplementary Fig. 3 LUBAC affects CENP-E localization on attached kinetochores. a Quantification of relative intensities of tested proteins at kinetochore on unaligned or aligned chromosomes in control or HOIP depleted mitotic cells. The relative intensity of each tested protein was normalized to its control cells. KT, kinetochore. **b** Immunoblots of HeLa cell lysates in **a** with the indicated antibodies. **c** Percentage of MG132-arrested mitotic cells with less than 50% of relative intensity of CENP-E at attached kinetochore on aligned chromosomes of control cells in control or HOIP depleted cells in Fig. 2g. **d** CENP-E localization in control or HOIL-1L and SHARPIN co-depleted mitotic cells synchronized by nocodazole or MG132. Green, CENP-E; red, CREST; blue, DNA. **e**, **f** Quantification of relative intensity of kinetochore CENP-E on unattached (**e**, Nocodazole treated, n = 18 cells in each group) or attached (**f**, MG132 treated, n = 17 cells in each group) kinetochores in (**d**). The average CENP-E/CREST ratio of each group was normalized to control cells. **g** CENP-E localization in Fig. 2j. Red, CENP-E; magenta, CREST; green, Myc-HOIP; blue, DNA. Data are presented as mean ± s.d.. NS, not significant, ***P < 0.001; two-sided Student's t-test. Dashed boxes, regions of aligned chromosomes. Scale bars, 5 μm.

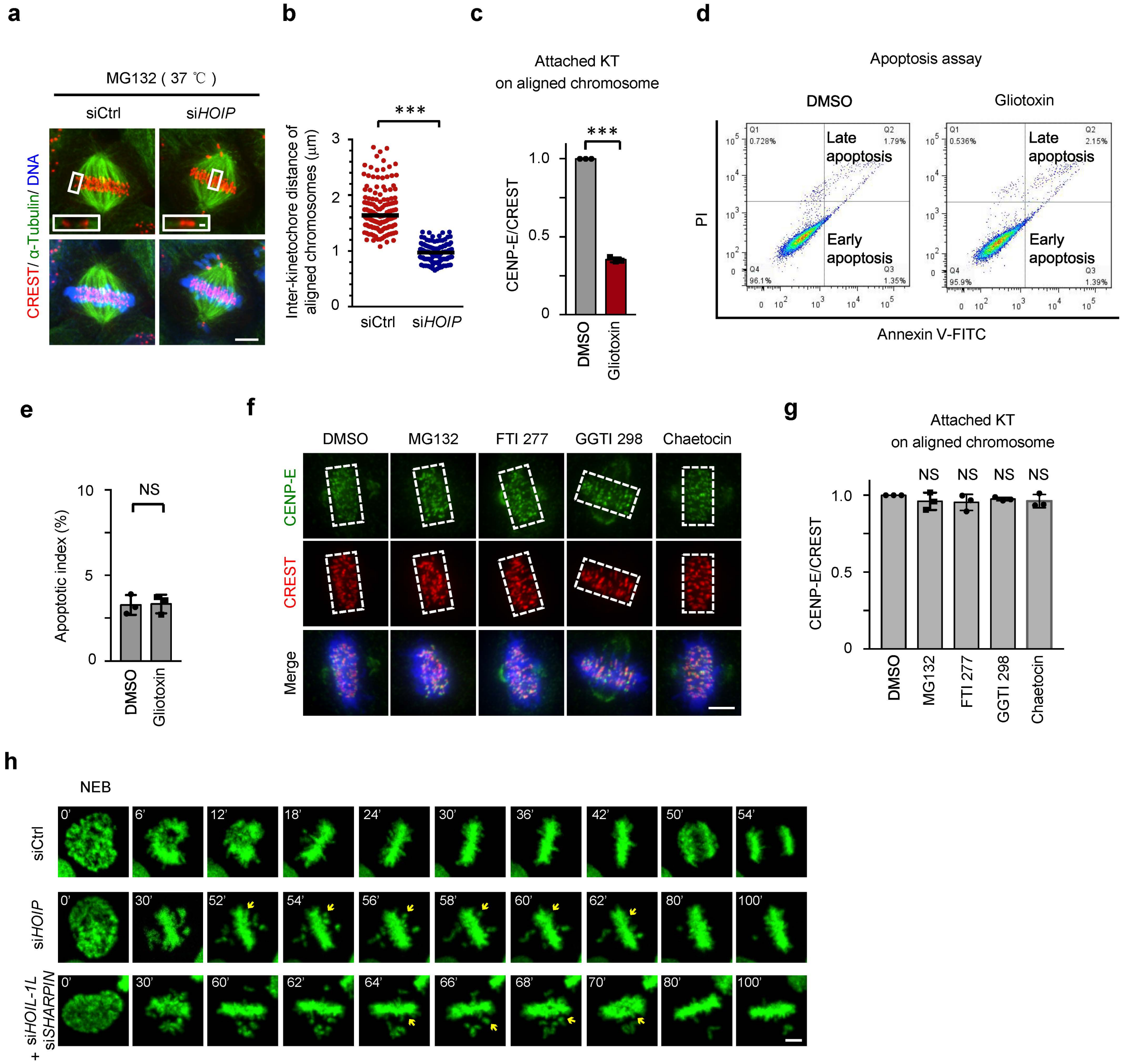

Supplementary Fig. 4 LUBAC regulates chromosome congression and alignment. a Immunofluorescence images of control or HOIP depleted mitotic cells synchronized into metaphase by MG132. Green, α-Tubulin; red, CREST; blue, DNA. Insets, zoomed-in views of the boxed regions. **b** Inter-kinetochore distances from cells in **(a)** were quantified by measuring the distance between sister kinetochores on aligned chromosomes. n = 10 cells in each group. Bars indicate the median. **c** Quantification of relative intensity of kinetochore CENP-E at attached kinetochores on aligned chromosomes in Fig. 3f, n = 18 cells in each group. The average CENP-E/CREST ratio of each group was normalized to control cells. **d** Analysis of apoptosis by FACS using Annexin V-FITC and Propidium Iodide

(PI). Velcade-arrested HeLa cells were released into medium containing DMSO or 1.2 μM Gliotoxin for 30 min, and then were collected and stained using an Annexin V-FITC Apoptosis Detection Kit (R&D systems). Early apoptosis: Annexin V-FITC positive, PI negative. Late apoptosis: Annexin V-FITC and PI double positive. **e** The proportion of apoptotic cells in DMSO or Gliotoxin treated cells in (**d**). **f** CENP-E kinetochore localization in HeLa cells treated with 100 nM Velcade for 2h, and then released into medium containing DMSO or different inhibitors for 30 min, including 10 μM MG132 (proteasome inhibitor), 10 μM FTI 277 (farnesyl transferase inhibitor), 10 μM GGTI 298 (GGTase inhibitor), or 1.3 μM Chaetocin (histone methyltransferases (G9a and SUV39H1) inhibitor). Green, CENP-E; red, CREST; blue, DNA. Dashed boxes, regions of aligned chromosomes. **g** Quantification of relative intensity of kinetochore CENP-E at attached kinetochores on aligned chromosomes in (**f**), $n = 16$ cells in each group. The average CENP-E/CREST ratio of each group was normalized to control cells. **h** High spatial and temporal resolution time-lapse imaging of control or LUBAC depleted HeLa/GFP-H2B cells. The numbers are minutes after NEB. Yellow arrows, chromosomes that lost alignment. Data are presented as mean ± s.d.. NS, not significant, $^{***}P < 0.001$; two-sided Student's *t*-test in **c**, **e** and **g**, two-sided Mann-Whitney *U* test in **b**. Scale bars, 5 μm (main image) and 0.5 μm (magnified region).

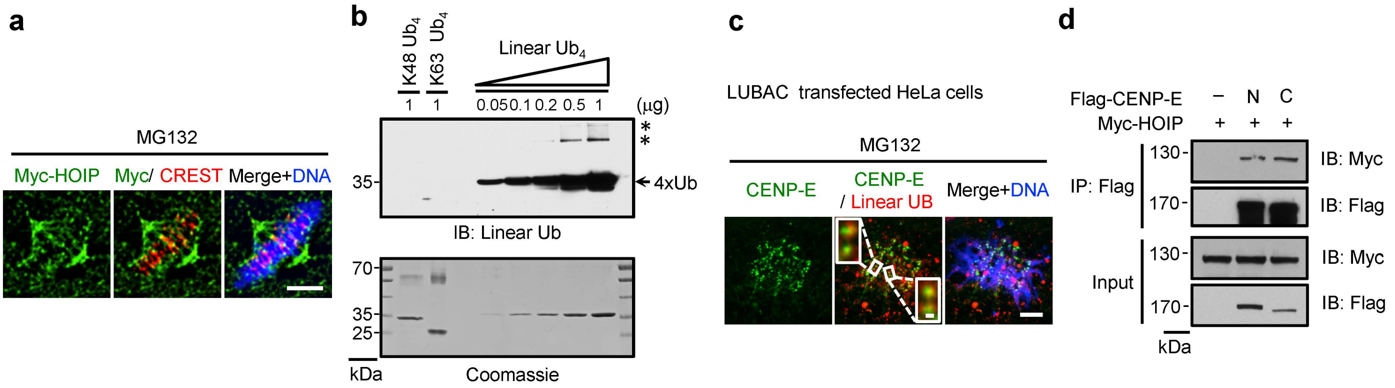

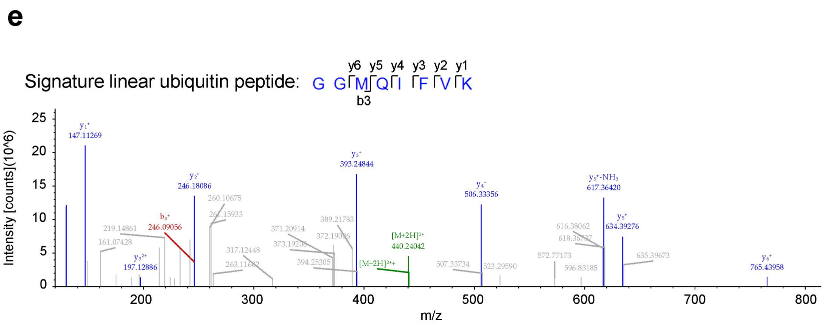

f

List of ubiquitinated sites and peptides of CENP-E:

Sites	Peptides
K45	TDNNVIYQVDGSk(GG)SFNFDR
K301	ILQNSLGGNAk(GG)TR
K346	NTPYVNEVSTDEALLk(GG)R
K546	k(GG)DQEMQLIHEISNLK
K580	HAEVYNQDLENELSSk(GG)VELLR

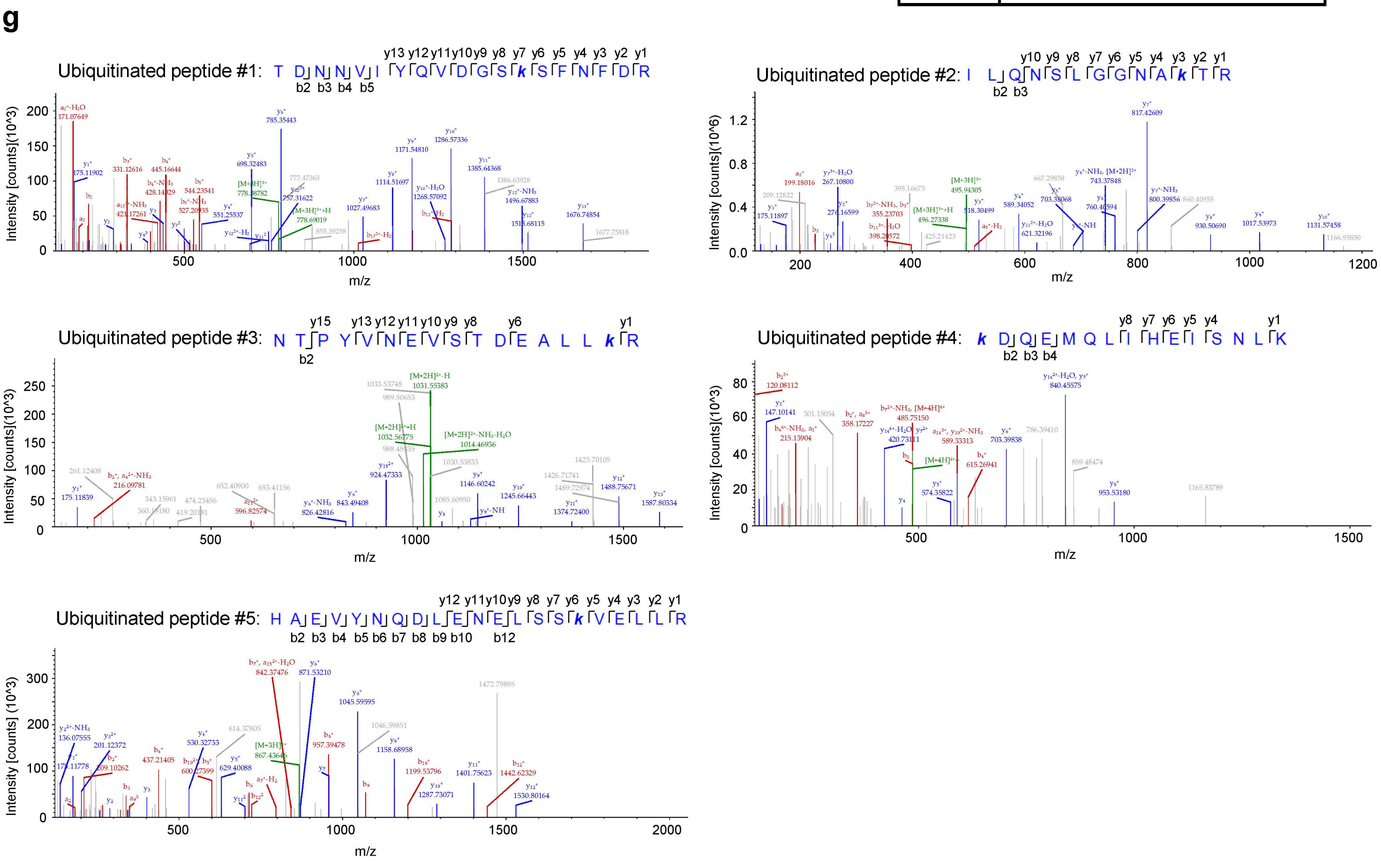

Supplementary Fig. 5 Identification of linear ubiquitinated sites of CENP-E. a Immunofluorescence images of exogenous HOIP detected with the Myc antibody in LUBAC overexpressed mitotic HeLa cells. Green, Myc-HOIP; red, CREST; blue, DNA. Scale bar, 2.5 μm. **b** Serial dilutions of linear-linked tetraUb

(0.05–1 μg) and 1 μg each of K48- or K63-linked tetraUb were immunoblotted with anti-linear Ub antibody (top), or Coomassie stained (bottom).‘*’ indicates linear-linked tetraUb that multimerizes. **c** Immunostaining of metaphase chromosome spread with the linear ubiquitin chain antibody and CENP-E antibody in LUBAC overexpressed mitotic HeLa cells. Insets, zoomed-in views of the boxed regions. Green, CENP-E; red, Linear UB; blue, DNA. Scale bars, 5 μm (main image) and 0.3 μm (magnified region). **d** The interaction between CENP-E truncations and ectopic HOIP in mitotic HEK293T cells. **e** GGMQIFVK is the peptide for linear linkage of ubiquitin. The MS/MS spectrum of the peptide for linear linkage of ubiquitin is shown. HEK293T cells were transfected with Flag-CENP-E (1-1360 aa), ubiquitin and LUBAC, polyubiquitylated Flag-CENP-E were immunoprecipitated and then subjected to Q Exactive HF mass spectrometer analysis. **f** The list of peptides containing ubiquitinated K45, K301, K346, K546, K580 of CENP-E identified by MS analysis. **g** The MS/MS spectrum of the individual peptides containing ubiquitinated K45, K301, K346, K546, K580 of CENP-E. Lowercase and italic ***k*** indicates K(GlyGly).

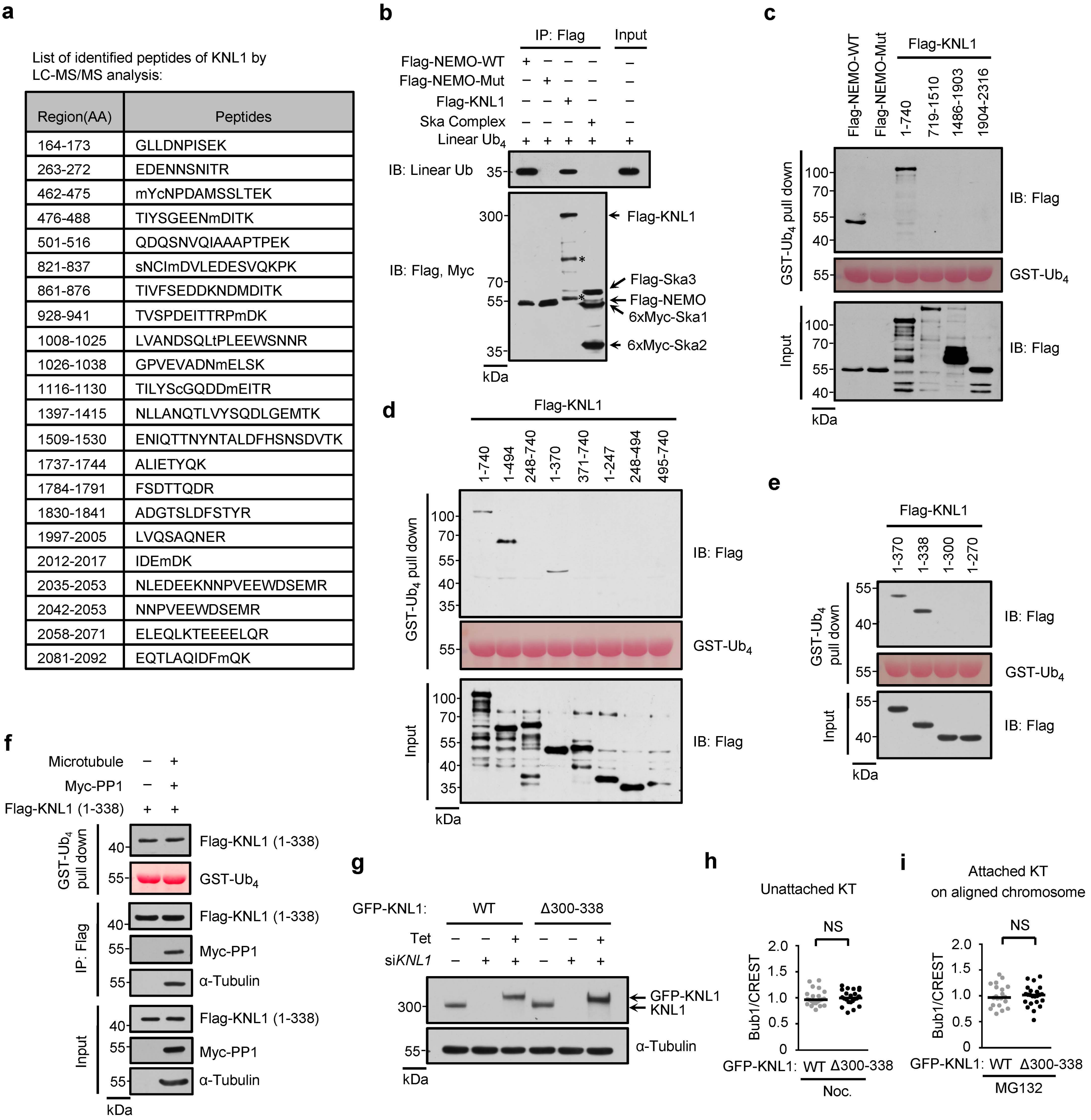

Region(AA)	Peptides
164-173	GLLDNPISEK
263-272	EDENNSNITR
462-475	mYcNPDAMSSLTEK
476-488	TIYSGEENmDITK
501-516	QDQSNVQIAAAPTPEK
821-837	sNCImDVLEDESVQKPK
861-876	TIVFSEDDKNDMDITK
928-941	TVSPDEITTRPmDK
1008-1025	LVANDSQLtPLEEWSNNR
1026-1038	GPVEVADNmELSK
1116-1130	TILYScGQDDmEITR
1397-1415	NLLANQTLVYSQDLGEMTK
1509-1530	ENIQTTNYNTALDFHSNSDVTK
1737-1744	ALIETYQK
1784-1791	FSDTTQDR
1830-1841	ADGTSLDFSTYR
1997-2005	LVQSAQNER
2012-2017	IDEmDK
2035-2053	NLEDEEKNNPVEEWDSEMR
2042-2053	NNPVEEWDSEMR
2058-2071	ELEQLKTEEEELQR
2081-2092	EQTLAQIDFmQK

Supplementary Fig. 6 KNL1 is the receptor of the linear ubiquitin chains. a Endogenous proteins pulled down from chromosome isolation fraction of MG132-arrested metaphase HeLa cells by GST-Ub4 were analyzed by LC-MS/MS. A list of identified peptides of KNL1 is shown. **b** Flag-tagged proteins were immunoprecipitated from MG312-arrest metaphase HEK293T cells by anti-Flag M2 affinity gel, and were incubated with the untagged linear ubiquitination chains in PDB buffer, then the M2 affinity gel were

washed five times with PDB buffer. The samples were detected by immunoblotting with anti-linear Ub antibody. One percent of purified Flag-tagged proteins were detected by western blot shown in the lower panel. ‘*’ indicates an unspecific band. **c**-**e** Flag-KNL1 truncations were translated in vitro and incubated with GST-Ub4 protein on GST-beads in PDB buffer, then GST-beads were washed and the sample were detected by immunoblotting with anti-Flag antibody. Loadings of GST proteins were determined by Ponceau S staining. **f** In vitro-translated Flag-KNL1N (1-338 aa) was pre-incubated with or without Myc-PP1 protein and taxol-stabilized microtubules, then this mixture was pulled down by GST-Ub4 on GST-beads or immunoprecipitated by anti-Flag affinity gel. **g** The knockdown efficiency of endogenous KNL1 and the expression of Tet-inducible GFP-KNL1 WT or GFP-KNL1Δ300-338 mutant in Fig. 6**i**-**k**. **h**, **i** Quantification of Bub1 signals on unattached (Nocodazole treated) (**h**, *n* = 22 cells in each group) or attached (MG132-treated) (**i**, GFP-KNL1 WT, *n* = 20 cells; GFP- KNL1Δ300-338, *n* = 23 cells) kinetochores in Flp-In T-REx HeLa stable cell lines depleted of endogenous KNL1. Each dot represents one cell. Bars indicate the median. NS, not significant; two-sided Student’s *t*-test.

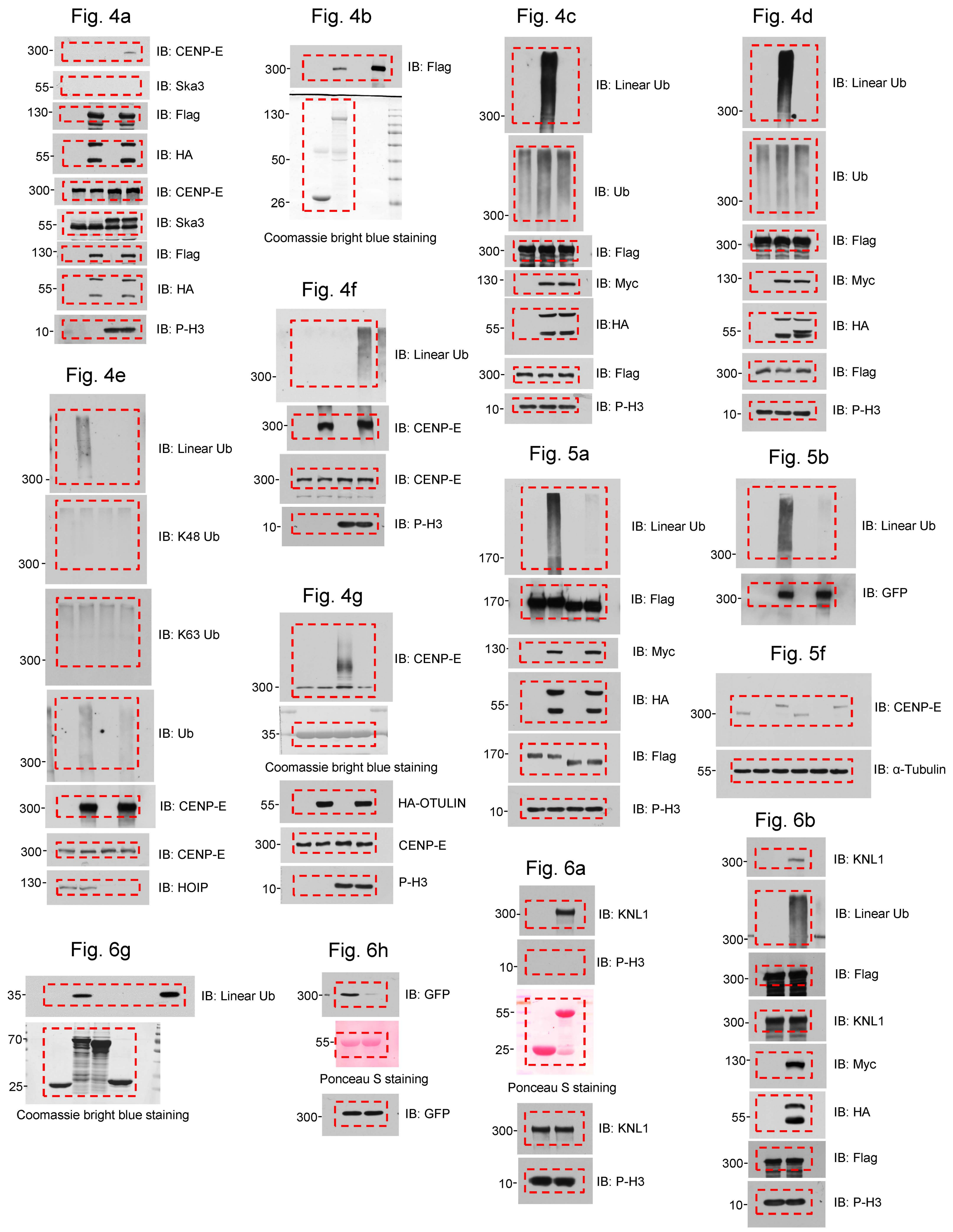

Fig. 4a
300- IB: CENP-E
55- IB: Ska3
130- IB: Flag
55- IB: HA
300- IB: CENP-E
55- IB: Ska3
130- IB: Flag
55- IB: HA
10- IB: P-H3
Fig. 4b
300- IB: Flag
130-
50-
26-
Coomassie bright blue staining
Fig. 4c
300- IB: Linear Ub
300- IB: Ub
300- IB: Flag
130- IB: Myc
55- IB:HA
300- IB: Flag
10- IB: P-H3
Fig. 4d
300- IB: Linear Ub
300- IB: Ub
300- IB: Flag
130- IB: Myc
55- IB: HA
300- IB: Flag
10- IB: P-H3
Fig. 4e
300 - IB: Linear Ub
300 - IB: K48 Ub
300 - IB: K63 Ub
300- IB: Ub
300- IB: CENP-E
300- IB: CENP-E
130- IB: HOIP
Fig. 4f
300- IB: Linear Ub
300- IB: CENP-E
300- IB: CENP-E
10- IB: P-H3
Fig. 4g
300- IB: CENP-E
35-
Coomassie bright blue staining
55- HA-OTULIN
300- CENP-E
10- P-H3
Fig. 5a
170- IB: Linear Ub
170- IB: Flag
130- IB: Myc
55- IB: HA
170- IB: Flag
10- IB: P-H3
Fig. 5b
300- IB: Linear Ub
300- IB: GFP
Fig. 5f
300- IB: CENP-E
55- IB: α-Tubulin
Fig. 6a
300- IB: KNL1
10- IB: P-H3
55-
25-
Ponceau S staining
300- IB: KNL1
10- IB: P-H3
Fig. 6b
300- IB: KNL1
300- IB: Linear Ub
300- IB: Flag
300- IB: KNL1
130- IB: Myc
55- IB: HA
300- IB: Flag
10- IB: P-H3
Fig. 6g
35- IB: Linear Ub
70-
25-
Coomassie bright blue staining
Fig. 6h
300- IB: GFP
55-
Ponceau S staining
300- IB: GFP

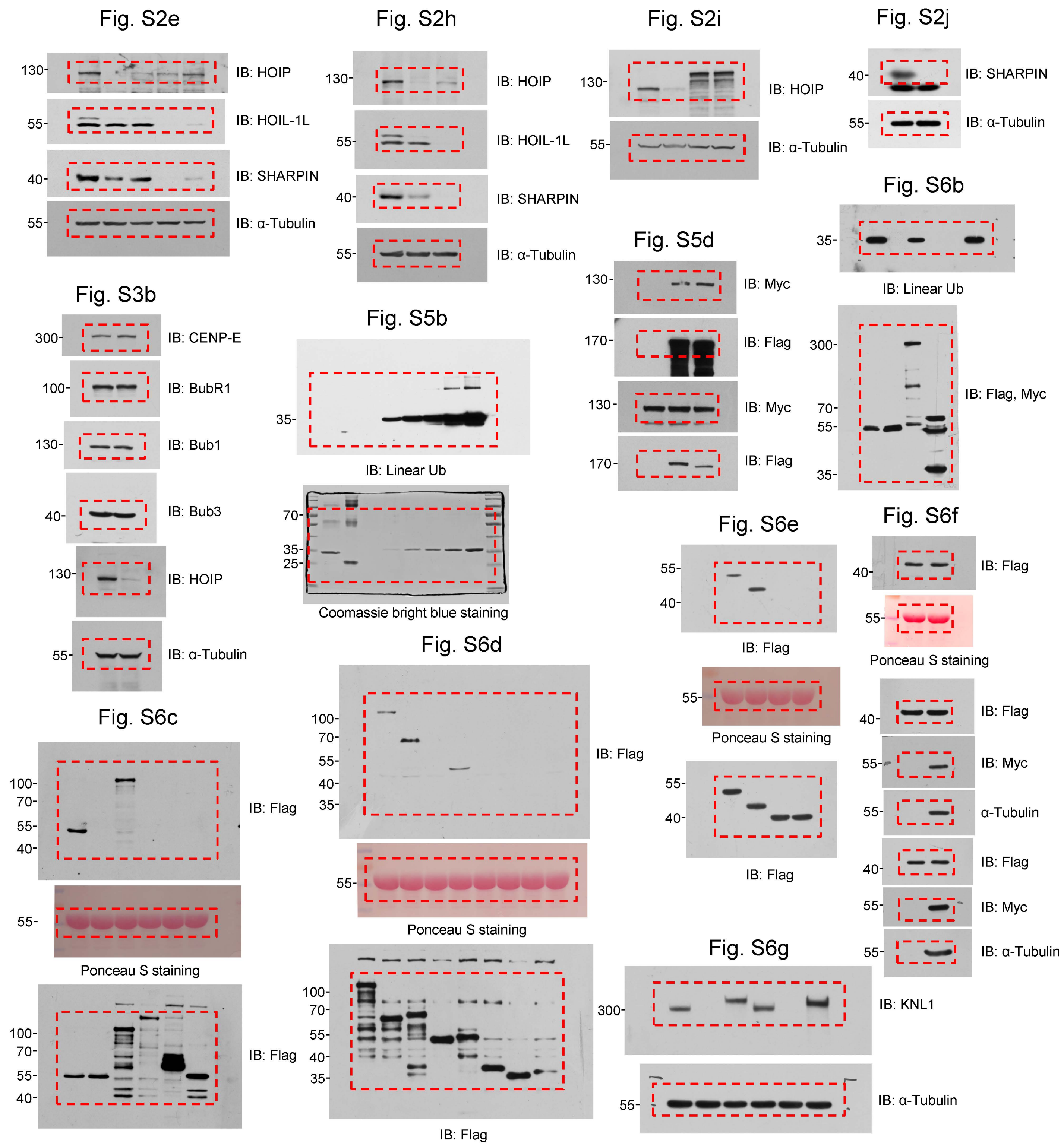

Supplementary Fig. 7 Unprocessed images of blots. Unprocessed images of scanned immunoblots shown in Figure 4,5,6 and Supplementary Figure 2, 3, 5, 6 are provided.
