# Supplementary material for: LUBAC controls chromosome alignment by targeting CENP-E to attached kinetochores
Source: Nat Commun. 2019 Jan 17;10:273. doi: 10.1038/s41467-018-08043-7 (PMC6336796; doi:10.1038/s41467-018-08043-7)
Supplement: Supplementary file 3 — Description of Additional Supplementary Files [file 41467_2018_8043_MOESM3_ESM.docx]

**Description of Additional Supplementary Files**

**File Name:** Supplementary Data 1

**Description:** Mass spectrometry data for identifying CENP-E ubiquitination sites.

**File Name:** Supplementary Data 2

**Description:** Mass spectrometry data for identifying GST-Ub4 interacting proteins.

**File Name:** Supplementary Data 3

**Description:** Sequences of siRNA and primers. The sequences of siRNAs, genotyping primers of cpdm mice and PCR primers used in this study.
