# Supplementary material for: LUBAC controls chromosome alignment by targeting CENP-E to attached kinetochores
Source: Nat Commun. 2019 Jan 17;10:273. doi: 10.1038/s41467-018-08043-7 (PMC6336796; doi:10.1038/s41467-018-08043-7)
Supplement: Supplementary file 2 — Peer Review File [file 41467_2018_8043_MOESM2_ESM.doc]

**Reviewers' comments:**

Reviewer #1 (Remarks to the Author):

This manuscript by Wu et al. describes the function of LUBAC and M1-linked Ub in the mitosis. A microscopy-based screen identified the LUBAC components HOIP, HOIL-1, and SHARPIN as important factors for chromosome alignment and mitotic progression. Mechanistically, the kinetochore motor CENP-E localizes to attached kinetochores in a LUBAC-dependent manner. This is mediated by binding of M1-ubiquitinated CENP-E by KNL1 at attached kinetochores, which mediates chromosome congression and alignment.

The authors have conducted a comprehensive analysis of the effects of LUBAC-deficiency on mitosis and investigated the molecular mechanisms underpinning the LUBAC-mediated mitotic progression. They identify a novel function of LUBAC, a new LUBAC substrate, as well as a new M1-linked Ub-binding protein. This work is very interesting and important to the field. The experiments presented in this manuscript are generally well performed and support the overall conclusions. 

1. Does LUBAC and M1-linked Ub localize to kinetochores? Does LUBAC interact directly with CENP-E? Or how does LUBAC find and ubiquitinate CENP-E?

2. From the experiments in 4a-c, the authors conclude that CENP-E is a substrate of LUBAC and are conjugated with M1-linked Ub. However, this cannot be concluded based on these experiments. By IP’ing on FLAG-tagged CENP-E and blotting for M1-linked Ub, the authors can only conclude that CENP-E is in a complex that contains M1-linked Ub. The true M1-substrate could be a different protein interacting with CENP-E that co-purifies in the IP. The mass spec data provided (supp Fig 5) does not directly show that CENP-E is a M1-Ub substrate either, just that there is M1-linked Ub in the IP and that CENP-E can be ubiquitinated (but that could be mono Ub or another chain type). To show that CENP-E is the M1-substrate, the authors could IP/pulldown on the M1-linked Ub, e.g. by using the M1-linked Ub antibody or affinity reagents such as the M1-SUB or M1-AP described in Fiil et al, Mol Cell 2013, PMID 23806334 and Draber et al, Cell Rep 2015, PMID 26670046, respectively (an M1 affinity reagent, M1-TUBE, is also commercially available from LifeSensors). After IP, they could blot for CENP-E. If the CENP-E blot shows a smear it can be concluded that CENP-E is ubiquitinated. If the smear can be reversed or reduced by OTULIN co-expression or addition of recombinant OTULIN after IP (see Emmerich et al, PNAS 2013, PMID 23986494 and Fiil et al, Mol Cell 2013, PMID 23806334) that would be good evidence for CENP-E being a substrate of M1-linked ubiquitination. See Emmerich and Cohen, Biochem Biophys Res Commun 2015 (PMID 26325464) for further details best practice and methods for studying ubiquitinated proteins.

3. In figs 4a-c (or figs that might replace them), it would be interesting to see a blot for total Ub and not just specific Ub chain types. This would inform on whether M1-linked Ub is the only chain type on CENP-E or whether other chain types are present as well. Rarely, M1-linked Ub chains occur alone on substrates. 

4. In their mass spec analysis of Ub chains in the CENP-E pulldown (sup fig 5b-c), do the authors find peptides from other Ub linkages than M1? 

5. To the best of my knowledge, gliotoxin is not a specific LUBAC inhibitor. It has been reported to inhibit LUBAC by prof. Kazuhiro Iwai’s lab (Sakamoto et al, ACS Chem Biol 2014), but that does not make it specific. Gliotoxin is also reported to inhibit or affect plethora of other processes and targets: it potently induces apoptosis in a number of cell types, it inhibits the proteasome, it inhibits farnesyl transferase and GGTase activity, and it affects histone methyltransferases. The authors should be careful about their interpretation of the gliotoxin experiment (Fig 3f) or consider an alternative approach.

6. The authors identify KNL1 as a putative M1-Ub-binding protein and how that KNL1 (and fragments thereof) expressed in cells are able to bind M1 tetraubiquitin. However, KNL1 has no reported or annotated domains for motifs that could obviously mediate this function and the authors do not provide evidence that this interaction is direct. Based on the experiments presented in fig5 and supp fig6 it cannot be excluded that it is a interactor of KNL1, which co-purifies in the IPs, that binds M1-Ub. The authors could consider expressing the minimal Ub-binding construct (1-338) recombinantly and show direct binding to 4xUb via an IP similar to fig 5g for instance.

Minor comments:

The authors state on page 6 in their results section: “Functions of LUBAC outside immunity has not been reported”. That is not correct. While LUBAC and M1-linked Ub, as far as we know, appears to mainly function in immunity and inflammation, functions have also been reported in DNA damage induced apoptosis and stress signaling (MacKay et al, Cancer Res 2014) and Wnt signalling (Rivkin et al, Nature 2013). The authors should rephrase their statement to reflect this.

The numbering on the Y-axis in fig 1a needs to be formatted correctly. 10 has been turned into a 0 and a 1.

From the authors’ model in Fig 6, it is unclear which protein is the Ub substrate and which is the Ub-binding protein. The authors might want to edit the model to reflect this better.

Reviewer #2 (Remarks to the Author):

In the manuscript by Wu et al, the authors suggest that the linear ubiquitin chain assembly complex (LUBAC) is involved in mitosis, and thus a potential anti-cancer target. To validate their findings, the authors use standard biochemical approaches in combination with mass spectrometric analysis to demonstrate that the kinetochore motor CENPE is targeted by linear ubiquitination.

The authors use an impressive amount of biochemical methods to further their hypothesis, but the current manuscript lacks direct evidence for the claims put forward. To address this, the authors use mass spectrometric analysis and report identification of several ubiquitination sites on CENPE, using the diGly remnant as an indicator for modified lysines. Unfortunately, the MS data does not look very convincing for several reasons: First, the authors report identification of diGly remnants residing on lysines located c-terminally in the identified peptides (Supplementary figure 5c). In here, ubiquitination of K1052 and K356 localize to c-terminally located lysines – which should not occur considering that ubiquitination should prevent tryptic digestion at these lysine residues due to sterical hindrance. Secondly, the authors used in-gel digestion for analyzing the CENPE protein, however, it has been demonstrated in the literature that this approach may cause mis-identification of precisely ubiquitination sites due to the usage of iodoacetamide (Nielsen et al, Nature Methods, 2008). Combined with the c-terminally located ubiquitination sites suggests that the observed ubiquitination sites may potentially just reflect in vitro artifacts due to the proteomic method used for the analysis. Furthermore, it is surprising that none of the reported sites listed in the manuscript has been identified in any of the many ubiquitination studies listed in the [phosphosite.org](http://phosphosite.org/) database. The authors would also have to explain this in detail.
Hence, the authors would have to redo the experiment in solution and completely avoid use of in gel digestion, and from this demonstrate that CENPe indeed becomes ubiquitinated in vivo.
Finally, the authors would have to make all the MS data available with their manuscript in order to evaluate the underlying spectra. Likewise, the authors should provide annotated MSMS spectra of the individual ubiquitination sites that they discover in their in solution analysis.

Additional questions related to the MS analysis, the authors use interaction proteomics for studying interactors of linear ubiquitination using GST-tetraubiqtuin. However, none of the acquired MS data seems to be included in the manuscript, except a list of identified peptide belonging to KNL1. Where these experiments performed in a quantitative manner, or just single analyses? The authors would have to provide compelling evidence that the identified and reported binding proteins of linear ubiquitination is not just proteins binding to the solid support, and similarly as mentioned above, the authors should make all the MS data available.

In conclusion, the current manuscript by Wu et al is not of a standard that would warrant publication in Nature communications.

Reviewer #3 (Remarks to the Author):

This extraordinarily well constructed manuscript establishes the mechanism through which the centromere/kinetochore motor protein CENP-E remains attached to kinetochores after initial microtubule attachment. Using a set of comprehensive approaches, the authors identify CENP-E as a substrate for addition of linear ubiquitin by the enzyme complex LUBAC and identify the KNL1 as the anchoring receptor for the linear ubiquitin changes. The effort is novel, comprehensive, and the evidence compelling. 

This reviewer provides ~50 reviews/year: this manuscript was at the top of those I have commented on in recent memory. It was a pleasure to read, the approaches chosen exactly the best approaches, and the experiments executed with unusual technical excellence. I strongly recommend publication. 

Minor comment: There is a formatting error in Figure 1a: the “10” unit on the vertical axis is split onto two lines.

**Point-to-point responses to reviewers’ comments:**

Reviewer #1

*This manuscript by Wu et al. describes the function of LUBAC and M1-linked Ub in the mitosis. A microscopy-based screen identified the LUBAC components HOIP, HOIL-1, and SHARPIN as important factors for chromosome alignment and mitotic progression. Mechanistically, the kinetochore motor CENP-E localizes to attached kinetochores in a LUBAC-dependent manner. This is mediated by binding of M1-ubiquitinated CENP-E by KNL1 at attached kinetochores, which mediates chromosome congression and alignment.*

*The authors have conducted a comprehensive analysis of the effects of LUBAC-deficiency on mitosis and investigated the molecular mechanisms underpinning the LUBAC-mediated mitotic progression. They identify a novel function of LUBAC, a new LUBAC substrate, as well as a new M1-linked Ub-binding protein. This work is very interesting and important to the field. The experiments presented in this manuscript are generally well performed and support the overall conclusions.*

*Reviewer 1 comment 1. Does LUBAC and M1-linked Ub localize to kinetochores? Does LUBAC interact directly with CENP-E? Or how does LUBAC find and ubiquitinate CENP-E?*

**Response:** To detect endogenous linear ubiquitin on kinetochores, we have tried to use different linear ubiquitin antibodies. We failed to detect any signal, presumably due to antibody insensitivity. To overcome this limitation, we transfected HeLa cells with the LUBAC complex to increase linear ubiquitin signals. We found linear ubiquitin indeed localized on kinetochores by immunostaining of metaphase chromosome spread with the anti-linear ubiquitin chain antibody (Matsumoto, M. L. et al., *J. Mol. Biol.,* 2012) (see revised Supplementary Fig. 5c). Similarly, we also failed to detect any signal of the endogenous LUBAC component using either commercial antibodies or antibodies we made in house. However, when we transfected LUBAC complex into HeLa cells, we were able to detect ectopically expressed HOIP on kinetochores (see revised Supplementary Fig. 5a).

We next investigated whether LUBAC can physically interact with CENP-E. We separately expressed Myc-HOIP and Flag-CENP-E in HEK293T cells and then purified each protein from mitotic cells. We performed the binding assay by incubating the eluted Flag-CENP-E with Myc-HOIP bound beads, and found that Myc-HOIP could bind to Flag-CENP-E protein *in vitro* (see revised Fig. 4b). Furthermore, we found that LUBAC interacted with CENP-E only in mitosis, but not interphase (see revised Fig. 4a). Consistently, the linear ubiquitination of endogenous CENP-E only occurs in mitosis (see revised Fig. 4f). Therefore, at least a pool of LUBAC localizes to kinetochores, physically interacts with CENP-E at these structures, and mediate the linear ubiquitination of CENP-E specifically during mitosis.

*Reviewer 1 comment 2. From the experiments in 4a-c, the authors conclude that CENP-E is a substrate of LUBAC and are conjugated with M1-linked Ub. However, this cannot be concluded based on these experiments. By IP’ing on FLAG-tagged CENP-E and blotting for M1-linked Ub, the authors can only conclude that CENP-E is in a complex that contains M1-linked Ub. The true M1-substrate could be a different protein interacting with CENP-E that co-purifies in the IP. The mass spec data provided (supp Fig 5) does not directly show that CENP-E is a M1-Ub substrate either, just that there is M1-linked Ub in the IP and that CENP-E can be ubiquitinated (but that could be mono Ub or another chain type). To show that CENP-E is the M1-substrate, the authors could IP/pulldown on the M1-linked Ub, e.g. by using the M1-linked Ub antibody or affinity reagents such as the M1-SUB or M1-AP described in Fiil et al, Mol Cell 2013, PMID 23806334 and Draber et al, Cell Rep 2015, PMID 26670046, respectively (an M1 affinity reagent, M1-TUBE, is also commercially available from LifeSensors). After IP, they could blot for CENP-E. If the CENP-E blot shows a smear it can be concluded that CENP-E is ubiquitinated. If the smear can be reversed or reduced by OTULIN co-expression or addition of recombinant OTULIN after IP (see Emmerich et al, PNAS 2013, PMID 23986494 and Fiil et al, Mol Cell 2013, PMID 23806334) that would be good evidence for CENP-E being a substrate of M1-linked ubiquitination. See Emmerich and Cohen, Biochem Biophys Res Commun 2015 (PMID 26325464) for further details best practice and methods for studying ubiquitinated proteins.*

**Response:** We appreciate the reviewer for pointing out this issue and giving us valuable suggestions. We learned a lot from these papers and cited these elegant papers in our revised manuscript (Fiil et al, *Mol. Cell,* 2013; Draber et al, *Cell Rep.,* 2015; Emmerich et al, *PNAS,* 2013; Emmerich et al, *Biochem. Biophys. Res. Commun.,* 2015). Following the reviewer’s suggestion, we have examined the linear ubiquitin of CENP-E with GST-coupled M1-SUB pull down assay. These data show that the linearly ubiquitinated CENP-E could only be detected in mitosis, but not in interphase (see revised Fig. 4g), which is consistent with the result of Fig. 4f in our revised manuscript. In addition, the smear above CENP-E can be reversed by OTULIN co-expression (see revised Fig. 4g). These results further support that CENP-E is a substrate of M1-linked polyubiquitination in mitosis.

*Reviewer 1 comment 3. In figs 4a-c (or figs that might replace them), it would be interesting to see a blot for total Ub and not just specific Ub chain types. This would inform on whether M1-linked Ub is the only chain type on CENP-E or whether other chain types are present as well. Rarely, M1-linked Ub chains occur alone on substrates.*

**Response:** The reviewer raised an important point. As suggested by the reviewer, we have redone the CENP-E linear ubiquitination assay. Similar to our original results, western blotting for M1 ubiquitin showed that CENP-E is a LUBAC substrate in mitosis. The blots for total Ub showed that the ubiquitination level of CENP-E was reduced when co-transfected with LUBAC ligase-dead CS mutant or the linear ubiquitin-specific deubiquitinase OTULIN (see revised Fig. 4c, 4d). However, the ubiquitin level of CENP-E is still detectable (see revised Fig. 4c, 4d). In addition, the total ubiquitination of endogenous CENP-E was reduced rather than abolished in HOIP-knockdown mitotic cells (see revised Fig. 4e). Thus, as the reviewer anticipated, these results suggested that M1-linked Ub might not be the only chain type on CENP-E in mitosis.

*Reviewer 1 comment 4. In their mass spec analysis of Ub chains in the CENP-E pulldown (sup fig 5b-c), do the authors find peptides from other Ub linkages than M1?*

**Response:** We appreciate the reviewer’s kind reminder. In mass spec analysis of Ub chains with CENP-E/LUBAC/Ub overexpressed cells, we indeed found peptides of other Ub linkages (K63, K48 and K11 Ub linkages) besides M1 linkages. We have revised manuscript accordingly and provided the complete MS dataset of the mass spec analyses of CENP-E ubiquitination sites (Table S1).

*Reviewer 1 comment 5. To the best of my knowledge, gliotoxin is not a specific LUBAC inhibitor. It has been reported to inhibit LUBAC by prof. Kazuhiro Iwai’s lab (Sakamoto et al, ACS Chem Biol 2014), but that does not make it specific. Gliotoxin is also reported to inhibit or affect plethora of other processes and targets: it potently induces apoptosis in a number of cell types, it inhibits the proteasome, it inhibits farnesyl transferase and GGTase activity, and it affects histone methyltransferases. The authors should be careful about their interpretation of the gliotoxin experiment (Fig 3f) or consider an alternative approach*.

**Response:** We thank the reviewer for raising this important issue. We completely agree with him/her. To rule out other effects of the inhibitor, we first detected the apoptosis of metaphase HeLa cells after Gliotoxin treatment (1.2 μM, 0.5 h) using the same condition as the original Fig. 3f, and found no increase in apoptosis (see revised Supplementary Fig. 4d and 4e). Next, to test whether the proteasome has an effect on CENP-E localization, we treated metaphase cells with the proteasome inhibitor MG132 (10μM, 0.5 h) and then detected the CENP-E localization on metaphase attached kinetochores. The result showed that CENP-E on aligned chromosomes at metaphase was unchanged in MG-132-treated cells (see revised Supplementary Fig. 4f and 4g), indicating it is unlikely that Gliotoxin regulates CENP-E localization through inhibiting the proteasome. Next, we detected the effect of inhibitors targeting farnesyl transferase (FTI 277), GGTase (GGTI 298) or histone methyltransferases (Chaetocin, G9a and SUV39H1 inhibitor) on CENP-E localization. Our data show that none of them have an effect on the localization of CENP-E at metaphase cells (see revised Supplementary Fig. 4f and 4g). Thus, according to the above results and our original data (Fig. 2 including siLUBAC and LUBAC-rescue experiments), we believe that Gliotoxin affects CENP-E localization at metaphase kinetochores likely through inhibiting LUBAC activity. Of course, we cannot exclude the possibility of the pan-inhibition of multiple known or unknown targets by Gliotoxin as a contributing factor to the observed phenotypes. We have revised our manuscript accordingly.

Additionally, based on previous studies (Nguyen, V. et al., *Mar. Drugs*, 2014; Kroll, M. et al., *Chemistry & Biology*, 1999; Vigushin, D.M. et al., *Medical Oncology*, 2004; Sun, Y. et al., *J. Nat. Prod.*, 2012), we hope to explain that the concentration of Gliotoxin we used is relatively low (1.2 μM) and the time is short (no more than 0.5 h). These conditions potentially minimize other effects of the drug in HeLa cells. We apologize for leaving out the time information of Gliotoxin treatment in our original manuscript and have included this in the figure legend of Fig. 3f in our revised manuscript.

*Reviewer 1 comment 6. The authors identify KNL1 as a putative M1-Ub-binding protein and how that KNL1 (and fragments thereof) expressed in cells are able to bind M1 tetraubiquitin. However, KNL1 has no reported or annotated domains for motifs that could obviously mediate this function and the authors do not provide evidence that this interaction is direct. Based on the experiments presented in fig5 and supp fig6 it cannot be excluded that it is a interactor of KNL1, which co-purifies in the IPs, that binds M1-Ub. The authors could consider expressing the minimal Ub-binding construct (1-338) recombinantly and show direct binding to 4xUb via an IP similar to fig 5g for instance.*

**Response:** We are grateful to the reviewer’s suggestion and performed the experiment accordingly. To detect direct binding of KNL1 to linear ubiquitin chains, we purified recombinant truncations of KNL1 (1-338) and performed *in vitro* GST pulldown assay. We found that 1-338 of KNL1, the minimal Ub-binding construct, could directly bind to the linear ubiquitin chains (see revised Fig. 6g). These data suggest that KNL1 is the direct receptor of linear ubiquitin chains.

*Reviewer 1 Minor comments:*

*The authors state on page 6 in their results section: “Functions of LUBAC outside immunity has not been reported”. That is not correct. While LUBAC and M1-linked Ub, as far as we know, appears to mainly function in immunity and inflammation, functions have also been reported in DNA damage induced apoptosis and stress signaling (MacKay et al, Cancer Res 2014) and Wnt signalling (Rivkin et al, Nature 2013). The authors should rephrase their statement to reflect this.*

**Response:** We thank the reviewer so much for pointing out this important issue and giving us a chance to correct our negligence. We have rephrased our statement accordingly and cited these two papers in our revised manuscript.

*The numbering on the Y-axis in fig 1a needs to be formatted correctly. 10 has been turned into a 0 and a 1.*

**Response:** Many thanks to the reviewer for his/her keen observation and suggestion. We have formatted the number on the Y-axis in Fig. 1a correctly in our revised manuscript (see revised Fig. 1a).

*From the authors’ model in Fig 6, it is unclear which protein is the Ub substrate and which is the Ub-binding protein. The authors might want to edit the model to reflect this better.*

**Response:** Thanks for the reviewer’s constructive suggestions. As suggested, we have revised our model to better distinguish the ubiquitinated substrate (bind to linear ubiquitin chain in a covalent way) and the ubiquitin binding protein (bind to linear ubiquitin chain in a non-covalent way) (see revised Fig. 7a).

Reviewer #2

*In the manuscript by Wu et al, the authors suggest that the linear ubiquitin chain assembly complex (LUBAC) is involved in mitosis, and thus a potential anti-cancer target. To validate their findings, the authors use standard biochemical approaches in combination with mass spectrometric analysis to demonstrate that the kinetochore motor CENPE is targeted by linear ubiquitination.*

*Reviewer 2 comment* *1. The authors use an impressive amount of biochemical methods to further their hypothesis, but the current manuscript lacks direct evidence for the claims put forward. To address this, the authors use mass spectrometric analysis and report identification of several ubiquitination sites on CENPE, using the diGly remnant as an indicator for modified lysines. Unfortunately, the MS data does not look very convincing for several reasons: First, the authors report identification of diGly remnants residing on lysines located c-terminally in the identified peptides (Supplementary figure 5c). In here, ubiquitination of K1052 and K356 localize to c-terminally located lysines – which should not occur considering that ubiquitination should prevent tryptic digestion at these lysine residues due to sterical hindrance. Secondly, the authors used in-gel digestion for analyzing the CENPE protein, however, it has been demonstrated in the literature that this approach may cause mis-identification of precisely ubiquitination sites due to the usage of iodoacetamide (Nielsen et al, Nature Methods, 2008). Combined with the c-terminally located ubiquitination sites suggests that the observed ubiquitination sites may potentially just reflect in vitro artifacts due to the proteomic method used for the analysis. Furthermore, it is surprising that none of the reported sites listed in the manuscript has been identified in any of the many ubiquitination studies listed in the phosphosite.org database. The authors would also have to explain this in detail.
Hence, the authors would have to redo the experiment in solution and completely avoid use of in gel digestion, and from this demonstrate that CENPe indeed becomes ubiquitinated in vivo.
Finally, the authors would have to make all the MS data available with their manuscript in order to evaluate the underlying spectra. Likewise, the authors should provide annotated MSMS spectra of the individual ubiquitination sites that they discover in their in solution analysis.*

**Response:** We appreciate the reviewer for pointing out this issue and giving us valuable suggestions. We took his/her comments seriously and carefully read the cited paper (Nielsen et al., *Nature Methods,* 2008). We redid the mass spectrometry experiments **in solution** according to Nielsen et al. In addition, to avoid the artifact caused by alkylating reagent iodoacetamide, we used chloroacetamide (a more selective alkylating reagent) during in-solution tryptic digestion. As the reviewer anticipated, peptides containing ubiquitinated K1052 or K356 could not be identified in this assay, indicating that ubiquitination of these two sites was likely caused by artifacts due to the use of iodoacetamide during in-gel digestion. However, we still identified peptides containing ubiquitinated K45, K301, K346, K546, K580 (see revised Supplementary Fig. 5f, 5g). We next established Flp-In T-REx HeLa stable cell lines expressing Tet-inducible wild type CENP-E or CENP-E 5KR mutant (K45R, K301R, K346R, K546R, K580R). The result shows that LUBAC-mediated linear ubiquitination of CENP-E 5KR mutant is dramatically decreased compared to wild-type CENP-E (see revised Fig. 5b). In addition, the CENP-E 5KR mutant also failed to localize to aligned chromosomes, but it retained localization on unaligned chromosomes (Fig. 5c-f). Thus, these five lysines sites are the major linear ubiquitination sites of CENP-E required for maintaining chromosome alignment, although we cannot exclude the possibility that the other sites have partial contributions to the linear ubiquitination of CENP-E because some other ubiquitination sites on CENP-E have been idenitified in this MS analysis. We have included the complete MS dataset of this new MS analysis in our revised manuscript (Table S1), and deposited the raw data to the PRIDE database (http://www.iprox.org/page/PSV023.html;?url=1538146981116SeMj with the password “tJiQ”). Accordingly, we provided the annotated MS/MS spectra of the linear ubiquitination site of CENP-E (see revised Supplementary Fig. 5g). Other individual ubiquitination sites of CENP-E are listed below (**MS/MS spectra for reviewer**). We sincerely thank the reviewer for this important suggestion, which has helped us avoid a potential mistake.

**MS/MS spectra for reviewer**

**Spectra #1:**

**Spectra #2:**

**Spectra #3:**

**Spectra #4:**

**Spectra #5:**

**Spectra #6:**

**Spectra #7:**

**Spectra #8:**

**Spectra #9:**

**Spectra #10:**

**Spectra #19:**

**Spectra #20:**

**Spectra #21:**

**Spectra #22:**

**Spectra #17:**

**Spectra #18:**

**MS/MS spectra for reviewer (continued)**

**Spectra #13:**

**Spectra #14:**

**Spectra #15:**

**Spectra #16:**

**Spectra #12:**

**Spectra #11:**

**Spectra #23:**

**Spectra #24:**

**Spectra #25:**

**Spectra #26:**

**MS/MS spectra for reviewer** The MS/MS spectrum of the other individual ubiquitination sites of CENP-E identified in solution analysis. Lowercase and [italic](https://cn.bing.com/dict/search?q=italic&FORM=BDVSP6&mkt=zh-cn) ***k*** indicates K(GlyGly).

The reviewer’s another concern is that “*it is surprising that none of the reported sites listed in the manuscript has been identified in any of the many ubiquitination studies listed in the phosphosite.org database.”* In our manuscript, we performed the MS to identify the linear ubiquitination sites on CENP-E using mitotic cells. In our revised manuscript, we further found that linear ubiquitination of endogenous CENP-E only occurs in mitosis, but not in interphase (see revised Fig. 4f, 4g). However, the reported CENP-E ubiquitination sites listed in the *phosphosite.org database* are mainly identified in asynchronous cells, which are mostly in interphase. We believe that the different cell cycle phases are most likely the reason why none of our identified sites have been listed in the *phosphosite.org database*.

*Reviewer 2 comment* *2. Additional questions related to the MS analysis, the authors use interaction proteomics for studying interactors of linear ubiquitination using GST-tetraubiqtuin. However, none of the acquired MS data seems to be included in the manuscript, except a list of identified peptide belonging to KNL1. Where these experiments performed in a quantitative manner, or just single analyses? The authors would have to provide compelling evidence that the identified and reported binding proteins of linear ubiquitination is not just proteins binding to the solid support, and similarly as mentioned above, the authors should make all the MS data available.*

**Response:** We thank the reviewer for the critical and constructive comments. In our original manuscript, we have enriched possible binding partners of linear ubiquitin chains on mitotic chromosomes from the isolated chromatin-containing fraction of metaphase HeLa cells using GST-tetraubiquitin, and then carried out mass spectrometry analysis. This MS analysis is not performed in a quantitative manner, and we have only done a single MS analysis. As the reviewer anticipated, we identified additional proteins in this MS analysis. Importantly, KNL1 had the highest score in our MS analysis. We have provided a supplemental table including a complete MS dataset in the revised manuscript (Table S2), and deposited the raw data to the PRIDE database (http://www.iprox.org/page/PSV023.html;?url=1538146981116SeMj with the password “tJiQ” ).

In addition, we used three different lines of evidence to demostrate that KNL1 directly binds to linear ubiqutin chains. First, we performed GST-vector pull down or GST-UB4 pull down assays. We incubated the GST-vector beads or GST-UB4 beads with cell lysates prepared from metaphase chromatin containing fraction of HeLa cells. We found that KNL1 only binds to GST-Ub4 beads, but not GST beads (see revised Fig. 6a). Second, we found that KNL1 purified from metaphase cells could efficiently bind to untagged linear tetraubiquitin chains (see revised Supplementary Fig. 6b). Third, *in vitro* GST pulldown assay showed that recombinant 1-338 of KNL1, but not 1-300 or 300-388 truncations, was able to bind to the linear ubiquitin chains (see revised Fig. 6g).

Taken together, we have provided strong evidence that KNL1 is a direct receptor for linear ubiquitin. Of course, we cannot exclude the existence of additional receptors, and have discussed this caveat. We hope the reviewer would agree that pursuing additional receptors is beyond the scope of this study.

Reviewer #3

*This extraordinarily well constructed manuscript establishes the mechanism through which the centromere/kinetochore motor protein CENP-E remains attached to kinetochores after initial microtubule attachment. Using a set of comprehensive approaches, the authors identify CENP-E as a substrate for addition of linear ubiquitin by the enzyme complex LUBAC and identify the KNL1 as the anchoring receptor for the linear ubiquitin changes. The effort is novel, comprehensive, and the evidence compelling.*

*This reviewer provides ~50 reviews/year: this manuscript was at the top of those I have commented on in recent memory. It was a pleasure to read, the approaches chosen exactly the best approaches, and the experiments executed with unusual technical excellence. I strongly recommend publication.*

*Minor comment: There is a formatting error in Figure 1a: the “10” unit on the vertical axis is split onto two lines.*

**Response:** We appreciate the reviewer’s encouraging comments on our manuscript. This reviewer kindly pointed out a formatting error, which has been corrected in our revised manuscript (see revised Fig. 1a).

**REVIEWERS' COMMENTS:**

Reviewer #1 (Remarks to the Author):

Wu and colleagues have done an extensive and beautiful job in revising this manuscript. I appreciate the length they went to strengthen the manuscript and their detailed rebuttal of points I and the other referees raised.

Particularly, I appreciate the numerous new and well-executed experiments the authors have performed to strengthen their conclusions on CENP-E being a LUBAC substrate and KNL1 being an M1-Ub-binding protein. All their new experiments support their initial findings and model and the result is a significantly stronger manuscript.

Overall, the authors have satisfactorily addressed the points I raised, and I think the revised manuscript is an exhaustive and carefully performed study of LUBAC's role in mitosis. This is an important new insight that will be a crucial contribution to the field. For these reasons, I think this study is well worth publication in Nature Communications.

Reviewer #2 (Remarks to the Author):

The authors have adequately addressed the comments raised.

minor additional comment:

- The uploaded RAW data is available on what seems a Chinese server (supported by the PRIDE database). However, when trying to access the server I'm faced with various security notifications and that the server is not safe. It would be recommended to move the raw data onto another server more easily accessible for the broad readers of Nature Communications.

Reviewer #3 (Remarks to the Author):

This reviewer had very much liked the initial version of this manuscript which claimed identification of the mechanism through which the centromere/kinetochore motor protein CENP-E remains attached to kinetochores after initial microtubule attachment. A set of comprehensive set of approaches was used to identify CENP-E as a substrate for addition of linear ubiquitin by the enzyme complex LUBAC and identify the KNL1 as the anchoring receptor for the linear ubiquitin changes. The effort is novel, comprehensive, and the evidence – in my view - was compelling.

Two other reviewers raised concerns about the compellingness of the mass spectrometry that formed the core of the efforts to identify linear ubiquitin bound to CENP-E. While this reviewer is not an expert in the corresponding methods, I found the reviewers’ concerns/criticisms to be sensible and required significant changes to produce a sufficiently compelling manuscript to recommend the work to Nat. Comm. In the revision the authors appear to have done just that, with a series of experimental extensions coupled with changes to the text.

The other two reviewers will no doubt assess whether their technical concerns have been adequately addresses, but for me the approaches have been improved and text is a pleasure to read. Pending opinions on the two other referees on the technical issues, I strongly recommend publication.

**Response to Reviewers’ comments:**

We have uploaded our mass spectrometry proteomics data to the ProteomeXchange Consortium to avoid any potential access issues raised by Reviewer #2. As noted below, the three reviewers had no further comments to address in revision. We appreciate the reviewers’ encouraging comments on our manuscript.

Reviewer #1:

*Wu and colleagues have done an extensive and beautiful job in revising this manuscript. I appreciate the length they went to strengthen the manuscript and their detailed rebuttal of points I and the other referees raised.*

*Particularly, I appreciate the numerous new and well-executed experiments the authors have performed to strengthen their conclusions on CENP-E being a LUBAC substrate and KNL1 being an M1-Ub-binding protein. All their new experiments support their initial findings and model and the result is a significantly stronger manuscript.*

*Overall, the authors have satisfactorily addressed the points I raised, and I think the revised manuscript is an exhaustive and carefully performed study of LUBAC's role in mitosis. This is an important new insight that will be a crucial contribution to the field. For these reasons, I think this study is well worth publication in Nature Communications.*

Reviewer #2:

*The authors have adequately addressed the comments raised.*

*minor additional comment:*

*- The uploaded RAW data is available on what seems a Chinese server (supported by the PRIDE database). However, when trying to access the server I'm faced with various security notifications and that the server is not safe. It would be recommended to move the raw data onto another server more easily accessible for the broad readers of Nature Communications.*

Reviewer #3:

*This reviewer had very much liked the initial version of this manuscript which claimed identification of the mechanism through which the centromere/kinetochore motor protein CENP-E remains attached to kinetochores after initial microtubule attachment. A set of comprehensive set of approaches was used to identify CENP-E as a substrate for addition of linear ubiquitin by the enzyme complex LUBAC and identify the KNL1 as the anchoring receptor for the linear ubiquitin changes. The effort is novel, comprehensive, and the evidence – in my view - was compelling.*

*Two other reviewers raised concerns about the compellingness of the mass spectrometry that formed the core of the efforts to identify linear ubiquitin bound to CENP-E. While this reviewer is not an expert in the corresponding methods, I found the reviewers’ concerns/criticisms to be sensible and required significant changes to produce a sufficiently compelling manuscript to recommend the work to Nat. Comm. In the revision the authors appear to have done just that, with a series of experimental extensions coupled with changes to the text.*

*The other two reviewers will no doubt assess whether their technical concerns have been adequately addresses, but for me the approaches have been improved and text is a pleasure to read. Pending opinions on the two other referees on the technical issues, I strongly recommend publication.*
